# Epidemiology of *Treponema pallidum*, *Chlamydia trachomatis*, *Neisseria gonorrhoeae*, *Trichomonas vaginalis*, and herpes simplex virus type 2 among female sex workers in the Middle East and North Africa: systematic review and meta-analytics

**DOI:** 10.7189/jogh.09.020408

**Published:** 2019-12

**Authors:** Hiam Chemaitelly, Helen A Weiss, Alex Smolak, Elzahraa Majed, Laith J Abu-Raddad

**Affiliations:** 1 Infectious Disease Epidemiology Group, Weill Cornell Medicine-Qatar, Cornell University, Qatar Foundation – Education City, Doha, Qatar; 2 MRC Tropical Epidemiology Group, London School of Hygiene and Tropical Medicine, London, United Kingdom; 3 Department of Infectious Disease Epidemiology, Faculty of Epidemiology and Population Health, London School of Hygiene and Tropical Medicine, London, United Kingdom; 4 Department of Healthcare Policy & Research, Weill Cornell Medicine, Cornell University, New York, New York, USA; 5 College of Health and Life Sciences, Hamad bin Khalifa University, Doha, Qatar

## Abstract

**Background:**

The epidemiology of sexually transmitted infections (STIs) and the role of commercial heterosexual sex networks in driving STI transmission in the Middle East and North Africa (MENA) region remain largely unknown.

**Objective:**

To characterize the epidemiology of *Treponema pallidum* (syphilis), *Chlamydia trachomatis*, *Neisseria gonorrhoeae*, *Trichomonas vaginalis*, and herpes simplex virus type 2 (HSV-2) among female sex workers (FSWs) in MENA using an in-depth quantitative assessment.

**Methods:**

A systematic review on ten international, regional, and country-level databases was conducted, and reported following PRISMA guidelines. Pooled prevalences of current and/or ever infection for each STI were estimated using random-effects meta-analyses. Sources of between-study heterogeneity were investigated through random-effects meta-regressions.

**Results:**

One *T. pallidum* incidence study and 144 STI prevalence studies were identified for 45 812 FSWs in 13 MENA countries. The pooled prevalence of current infection was 12.7% (95% confidence interval (CI) = 8.5%-17.7%) for *T. pallidum*, 14.4% (95% CI = 8.2%-22.0%) for *C. trachomatis*, 5.7% (95% CI = 3.5%-8.4%) for *N. gonorrhoeae*, and 7.1% (95% CI = 4.3%-10.5%) for *T. vaginalis*. The pooled prevalence of ever infection (seropositivity using antibody testing) was 12.8% (95% CI = 9.4%-16.6%) for *T. pallidum*, 80.3% (95% CI = 53.2%-97.6%) for *C. trachomatis*, and 23.7% (95% CI = 10.2%-40.4%) for HSV-2. The multivariable meta-regression for *T. pallidum* infection demonstrated strong subregional differences, with the Horn of Africa and North Africa showing, respectively 6-fold (adjusted odds ratio (AOR): 6.4; 95% CI = 2.5-16.7) and 5-fold (AOR = 5.0; 95% CI = 2.5-10.6) higher odds of infection than Eastern MENA. There was also strong evidence for declining *T. pallidum* odds of infection at 7% per year (AOR = 0.93; 95% CI = 0.88-0.98). Study-specific factors including diagnostic method, sample size, sampling methodology, and response rate, were not associated with syphilis infection. The multivariable model explained 48.5% of the variation in *T. pallidum* prevalence.

**Conclusions:**

STI infection levels among FSWs in MENA are considerable, supporting a key role for commercial heterosexual sex networks in transmission dynamics, and highlighting the health needs of this neglected and vulnerable population. Syphilis prevalence in FSWs appears to have been declining for at least three decades. Gaps in evidence persist for multiple countries.

The burden of sexually transmitted infections (STIs) and sequelae remains a major global health concern [1]. Nearly one million persons are infected with a curable STI every day [2], and about half a billion are living with herpes simplex virus type 2 (HSV-2) [3]. The largely asymptomatic nature of STIs, particularly for women, leaves most individuals unaware of their infection [1]. STIs have been associated with HIV acquisition [4-6], and poor reproductive health outcomes including pelvic inflammatory disease, ectopic pregnancy, infertility, and perinatal deaths [1,7].

Commercial heterosexual sex networks (CHSNs) are believed to play a critical role in STI transmission [8-10]. STIs have been demonstrated as proxy biomarkers of sexual risk behaviour [11,12], and as a powerful tool for understanding the structure of sexual networks and predicting HIV epidemic potential [11-13]. However, unlike HIV, STI epidemiology in CHSNs remains, globally, a neglected area of research [1]. Programmatically, STI surveillance among female sex workers (FSWs) continues to be weak and infection levels poorly quantified [1]. Sexual propagation of STIs along CHSNs is also poorly understood given the dearth or limited validity of self-reported sexual behaviour data [13-15].

To attend to the United Nations’ Sustainable Development Goals (SDGs) and targets [16], particularly SDG3 target of “ensuring universal access to sexual and reproductive health services” [16], and to reduce the global burden of disease attributed to STIs, the World Health Organization (WHO) has recently formulated the “Global Health Sector Strategy on STIs” [6]. The goal of this strategy is to eliminate STIs as a major public health concern by 2030 through an integrated approach for prevention and control [6]. Milestones for 2020 include achieving 70% coverage for comprehensive STI prevention services among key populations [6]. The strategy’s first strategic direction entails “understanding the STI epidemic as a basis for advocacy, political commitment, national planning, resource mobilization and allocation, implementation, and programme improvement” [6].

Despite remarkable progress in HIV research [17], and an understanding of the role of FSWs [18], people who inject drugs (PWID) [19], and men who have sex with men (MSM) [20], in the HIV epidemic in the Middle East and North Africa (MENA) region, the epidemiology of STIs and the role of CHSNs in driving STI transmission remain largely unknown [21]. The two global reviews of STI epidemiology in FSWs had no data for any of the 23 MENA countries [22,23]. A large volume of STI data in the region resides in databases that were never analyzed, or in country-level reports that were never published in the scientific literature [24,25].

Against this background, our study aimed to characterize the epidemiology of key STIs among FSWs in MENA by 1) systematically reviewing and synthesizing all available published and unpublished evidence for *Treponema pallidum* (henceforth referred to as syphilis), *Chlamydia trachomatis*, *Neisseria gonorrhoeae*, *Trichomonas vaginalis*, and HSV-2 incidence and/or prevalence, 2) estimating, for each STI, the pooled mean prevalence of current and/or ever (seropositivity using antibody testing) infection, and 3) identifying sources of between-study heterogeneity, and regional and temporal trends associated with STI prevalence.

## METHODS

We conducted a systematic review and an in-depth quantitative assessment to characterize STI epidemiology among FSWs in MENA. Details of the study methodology (including specific statistical analyses) can be found in subsequent sections.

### Search strategy and selection criteria

Evidence for syphilis, *C. trachomatis*, *N. gonorrhaoeae*, *T. vaginalis*, and HSV-2 immunoglobulin G (IgG) incidence and/or prevalence among FSWs in MENA was systematically reviewed, informed by Cochrane’s Collaboration guidelines [26]. Findings were reported following Preferred Reporting Items for Systematic Reviews and Meta-analyses (PRISMA) guidelines [27] (checklist in Table S1 in **Online Supplementary Document**). The MENA definition covers 23 countries—Afghanistan, Algeria, Bahrain, Djibouti, Egypt, Iran, Iraq, Jordan, Kuwait, Lebanon, Libya, Morocco, Oman, Pakistan, Palestine, Qatar, Saudi Arabia, Somalia, Sudan, Syria, Tunisia, United Arab Emirates (UAE), and Yemen—based on convention in HIV research [19,20,24,25], and definitions of WHO, Joint United Nations Programme on HIV/AIDS (UNAIDS), and World Bank [24].

Systematic searches were performed up to September 20, 2018, on international databases (PubMed and Embase), regional and national databases (WHO Global Health Observatory data repository [28], WHO African Index Medicus database, WHO Index Medicus for the Eastern Mediterranean Region database, Iranian Scientific Information Database, Iraqi Academic Scientific Journals’ database, and Pakistan’s PakMediNet database), abstract archives of International AIDS Society Conferences [29], as well as published and unpublished country-level and international organizations’ reports available through the MENA HIV/AIDS Epidemiology Synthesis Project database [24,25]. Search strings were broad (MeSH/Emtree terms exploded to cover all subheadings and free text terms) with no language or year restrictions (Box S1 in **Online Supplementary Document**).

Duplicate citations were identified using a reference manager, Endnote. Titles and abstracts were then screened for relevance, with relevant/potentially relevant citations undergoing full-text screening. Any document reporting an incidence and/or prevalence measure in FSWs for an STI of interest, based on primary data, was eligible for inclusion. Case reports, case series, editorials, commentaries, and reviews were excluded. Hand searching was further performed on reference lists of all relevant articles.

The term *‘study’* is used here to refer to a specific STI incidence or prevalence measure in a specific FSW population. Accordingly, one document/report could contribute multiple studies and one study could be published in different reports. Duplicate study results were included only once using the more detailed/recent report.

### Data extraction and synthesis

Extraction was performed by HC, and double extraction by AS (extraction list in Box S2 in **Online Supplementary Document**). Discrepancies were settled by consensus, or by contacting authors. Full-texts in languages other than English were extracted by native speakers. Data were stratified by infection type (current vs ever (seropositivity using antibody testing)), and summarized using medians, ranges, and interquartile ranges (IQR). Definitions of infection types and details of the classification of diagnostic methods’ results into current, recent, and ever infection can be found in Table S2 in **Online Supplementary Document**. It was assumed, for *N. gonorrhoeae* and *T. vaginalis* studies, whenever a diagnostic method was not explicitly specified, that the diagnostic method assessed current infection.

All STI studies were extracted and reported. However, studies applying the same assay to different biological specimens from the same person were included only once in analyses, for statistical independence. This was done based on a sequential order that prioritized infection detection in endocervical swabs, followed by vaginal, then urine samples. Studies assessing prevalence using different diagnostic methods, were also included only once in analyses, with studies using polymerase chain reaction prioritized over those using culture or other methods.

### Quality assessment

The quality assessment for each STI prevalence study was informed by Cochrane Collaboration guidelines (criteria in Table S3 in **Online Supplementary Document**) [30]. Studies were classified as having “low” vs “high” risk of bias (ROB) on each of three quality domains assessing the 1) rigor of sampling methodology (probability-based; non-probability-based), 2) response rate (≥60% or ≥60% of target sample size reached for studies using respondent-driven or time-location sampling; <60%), and 3) STI ascertainment (biological assay explicitly indicated; otherwise). Studies with missing information for a specific domain were classified as having “unclear” ROB for that domain.

Given reported limitations in HSV-2 diagnostics [31,32], the quality of HSV-2 assays was determined by consulting with an expert advisor, Professor Rhoda Ashley-Morrow, University of Washington, Seattle. Studies where the validity of the diagnostic method could not be confirmed, were excluded from the systematic review.

Quality domains were included in meta-regression analyses (described below) to assess their impact on prevalence.

### Meta-analyses

For each STI, the pooled mean prevalence of current and/or ever infection, along with the corresponding 95% confidence intervals (CIs), were estimated using meta-analysis. Overall prevalence measures were replaced by their strata where applicable. For each study, one final stratification was considered based on a pre-defined sequential order that prioritizes country of origin, followed by type of FSW, year, region, and age. Subregional and time-trend analyses were conducted as warranted by data. Variances were stabilized using Freeman-Tukey type arcsine square-root transformation [33,34]. Weights were applied using the inverse-variance method [34,35], before pooling measures using a Dersimonian-Laird random-effects model [36], thereby accounting for sampling variation and for true heterogeneity [37]. Missing sample sizes for measures or their strata (<4% of all studies) were imputed using the median sample size, as calculated from studies with available information.

Heterogeneity assessment used Cochran’s Q statistic to confirm existence of heterogeneity across studies, I^2^ to determine magnitude of between-study variation that is due to true differences in effect size (prevalence) rather than chance, and prediction intervals to estimate the 95% interval of the true effect sizes’ distribution [37,38].

Meta-analyses were implemented in R 3.4.2 (R core team, Vienna, Austria) [39].

### Meta-regressions

Only syphilis had a considerable number of measures (>100) to warrant conduct of random-effects meta-regression analyses. Independent variables considered *a priori* were: country/subregion, year of data collection, infection type, diagnostic method, STI ascertainment, sample size, sampling methodology, and response rate. Details of subgrouping and justifications are in Table S4 in **Online Supplementary Document**. Meta-regression was conducted using the log-transformed odds of syphilis infection and corresponding variance. Factors associated with higher odds of infection at *P* ≤ 0.10 in univariable analyses were included in the multivariable analysis. Factors with *P* ≤ 0.05 in the multivariable model were considered as significant predictors of heterogeneity in syphilis prevalence.

Meta-regressions were implemented in Stata/SE 15.1 (StataCorp, College Station, TX, USA) [40].

## RESULTS

### Search results and scope of evidence

**Figure 1:**
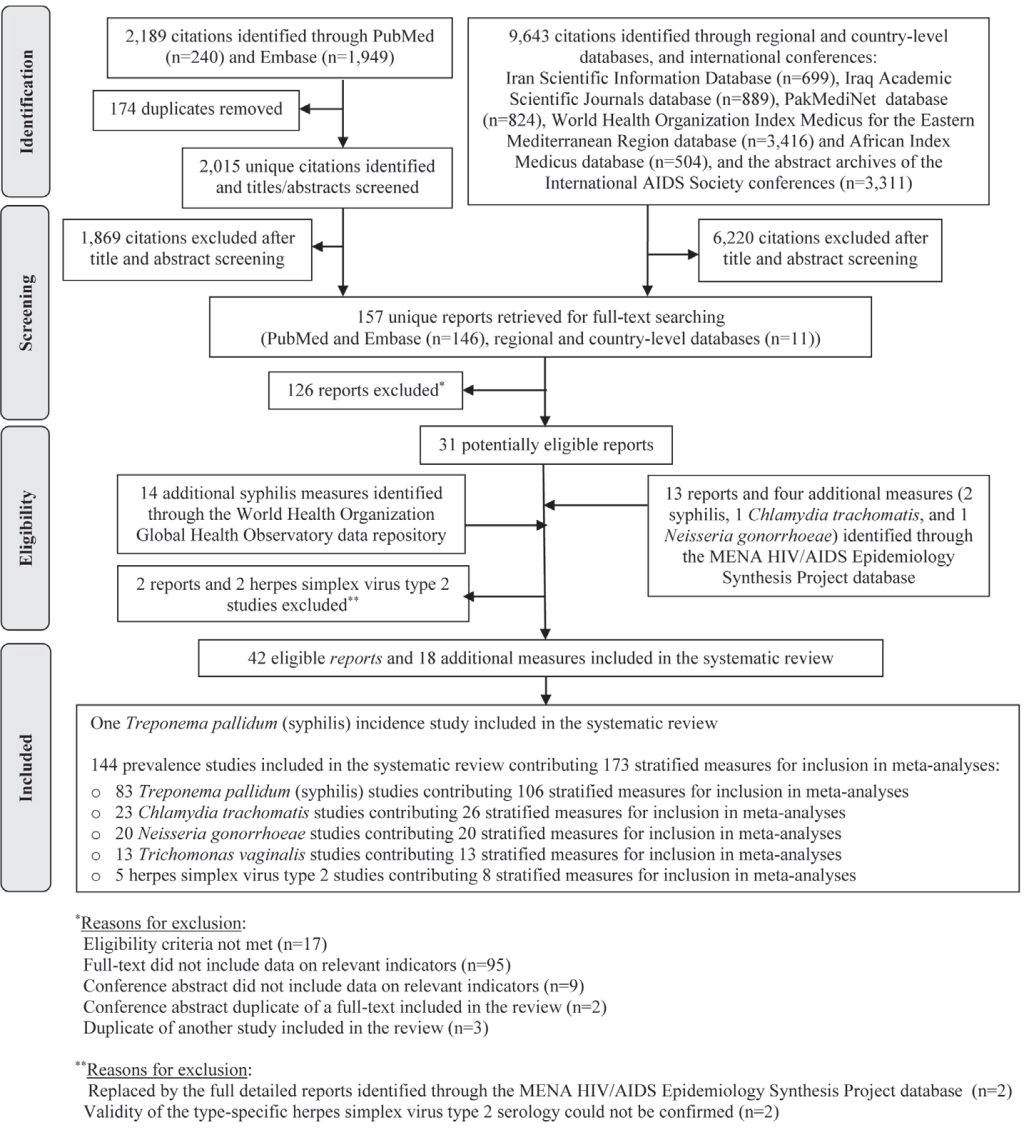
Flowchart presenting the process of study selection following PRISMA guidelines [27].

**Figure 1** shows the study selection process based on PRISMA. The search identified a total of 11 832 citations: 240 through PubMed, 1949 through Embase, and 9643 through the rest of the databases. After removing duplicates and screening of titles and abstracts, 157 reports qualified for full-text screening, of which 31 were eligible for inclusion in the systematic review.

Thirteen additional reports, two of which replaced eligible articles, and four additional STI measures, were further identified through the MENA HIV/AIDS Epidemiology Synthesis Project database. Fourteen additional syphilis prevalence measures were identified through the WHO Global Health Observatory data repository. Two studies were excluded based on consultation with Professor Rhoda Ashley-Morrow, an expert advisor in HSV-2 diagnostics, because the validity of the type-specific HSV-2 serology could not be confirmed [41,42].

In sum, 42 eligible reports and 18 additional STI measures were included in the systematic review. These yielded one syphilis incidence study, and 144 prevalence studies assessing the different STIs. The latter contributed 173 stratified measures for inclusion in meta-analyses and meta-regressions.

STI prevalence data were available for 45 812 FSWs from 13 of the 23 MENA countries. Nearly two-thirds (58.9%) of prevalence studies assessed syphilis (in 29 769 FSWs), 16.3% assessed *C. trachomatis* (in 5613 FSWs), 12.8% assessed *N. gonorrhoeae* (in 5230 FSWs), 8.5% assessed *T. vaginalis* (in 4258 FSWs), and 3.6% assessed HSV-2 IgG (in 942 FSWs). Most studies (80.8%) were conducted post-2000. Over half (51.1%) of studies reported on current infection, 30.5% on ever infection (seropositivity using antibody testing), and 1.4% on recent infection. Time of exposure was unclear for the rest of studies (17.0%).

### Incidence studies

The only one identified incidence study assessed syphilis incidence in FSWs. The study was conducted in 1988 in Mogadishu, Somalia, and reported cumulative incidence at 12.5% after six months of follow-up [43].

### Prevalence studies

Prevalence of current syphilis infection among FSWs ranged, across studies (n = 28), from 0%-50.8%, with a median of 9.4% (IQR = 3.0%-23.4%; **Table 1**). Meanwhile, seropositivity for syphilis (n = 33) antibodies ranged from 0%-69.0%, with a median of 4.2% (IQR = 1.9%-15.2%).

**Table 1:** Prevalence of syphilis among FSWs in the Middle East and North Africa*

Country short citation	Year(s) of data collection	City/province	Sampling	Study site	Assay type	Tested (n)	Prevalence (%)
**CURRENT INFECTION**							
**Afghanistan:**							
Todd, 2010 [44]	2006-08	Jalalabad, Kabul, Mazar-i-Sharif	Conv	NGO	RPR+ & TPHA+	520	0
**Egypt:**							
MOH, 2000 [45]	1999-00	Greater Cairo	Conv	Community	RPR+ & TPHA+	52	5.8
**Iran:**							
Kassaian, 2012 [46]	2009-10	Isfahan	Conv	Prison, drop-in center	RPR+	91	0
Navadeh, 2012 [42]	2010	Kerman	RDS	Community	VDRL+	139	7.2
Kazerooni, 2014 [41]	2010-11	Shiraz	RDS	Community	VDRL+ & FTA-ABS+	278	0
Jahanbakhsh, 2017 [47]	2012	Tehran	Conv	Homeless shelters	RPR+	14	0
**Morocco:**							
MOH, 2008 [48]	2007	Agadir, Rabat-Sale, Tanger	Conv	Clinic	VDRL+ & TPHA+	141	13.5
MOH, 2012 [49]	2011-12	Agadir	RDS	Community	VDRL+ & TPHA+	362	21.4
MOH, 2012 [49]	2011-12	Fes	RDS	Community	VDRL+ & TPHA+	359	18.8
MOH, 2012 [49]	2011-12	Rabat	RDS	Community	VDRL+ & TPHA+	392	13.9
MOH, 2012 [49]	2011-12	Tanger	RDS	Community	VDRL+ & TPHA+	318	13.3
**Pakistan:**							
Baqi, 1998 [50]	1993-94	Karachi	Conv	Red-light district	VDRL+ & FTA-ABS+	81†	5.0
Rehan, 2009 [51] & NACP, 2005 [52]	2004	Karachi	Snowball	Community	RPR+ & TPHA+	421	3.6
Rehan, 2009 [51] & NACP, 2005 [52]	2004	Lahore	SyCS	Red-light district	RPR+ & TPHA+	387	16.0
Shah, 2004 [53]	2004	Hyderabad	Conv	Community	VDRL+ & TPHA+	157	11.5
Hawkes, 2009 [54]	2007	Abbottabad	RDS	Community	RPR+ & TPHA+	107	2.8
Hawkes, 2009 [54]	2007	Rawalpindi	RDS	Community	RPR+ & TPHA+	426	1.2
Khan, 2011 [55]	2007	Lahore	RDS	Community	RPR+ & TPHA+	730	4.5
**Somalia:**							
Jama, 1987 [56]	1985-86	Mogadishu	Conv	Community	VDRL+ & TPHA+	85	44.7
Jama Ahmed, 1991 [43]	1988-89	Mogadishu	Conv	Community	VDRL/RPR+ & TPHA+	155	47.7
Scott, 1991 [57]	1989	Kismayu, Merca	Conv	NR	RPR+ & FTA-ABS+	57	50.8
Corwin, 1991 [58]	1990	Chismayu, Merca, Mogadishu	Conv	NR	RPR+ & FTA-ABS+	302	35.4
Watts, 1994 [59]	1990	Chismayu, Merca, Mogadishu	Conv	NR	RPR+ & FTA-ABS+	236	30.9
IOM, 2017 [60]	2014	Hargeisa	RDS	Community	RDT+ & RPR+	96	2.4
**Sudan:**							
MOH, 2016 [61]	2015-16	Juba, South Sudan	RDS	Community	RDT+ & RPR+	832	7.3
**Tunisia:**							
Bchir, 1988 [62]	1987	Sousse	Conv	NR	VDRL+ & TPHA+	42	28.6
Ayachi, 1997 [63]	1992-94	Tunis	Conv	NR	VDRL+ & TPHA+	79	24.1
**Yemen:**							
Stulhofer, 2008 [64]	2008	Aden	RDS	Community	VDRL+	244	4.9
**EVER INFECTION‡**							
**Afghanistan:**							
NACP, 2010 [65]	2009	Kabul	RDS	Community	RDT+	368	5.4
NACP, 2012 [66]	2012	Herat	RDS	Community	RDT+	344	0.9
NACP, 2012 [66]	2012	Kabul	RDS	Community	RDT+	333	0.0
NACP, 2012 [66]	2012	Mazar-i-Sharif	RDS	Community	RDT+	355	2.0
**Algeria:**							
MOH, 2009 [67]	2004	National	Conv	Sentinel surveillance	TPHA+	185	11.9
MOH, 2009 [67]	2007	National	Conv	Sentinel surveillance	TPHA+	380	18.4
**Iran:**							
Mirzazadeh, 2016 [68]	2015	National	Conv	Community, clinic	RDT+	1,337	0.4
**Pakistan:**							
Hawkes, 2009 [54]	2007	Abbottabad	RDS	Community	TPHA+	107	2.8
Hawkes, 2009 [54]	2007	Rawalpindi	RDS	Community	TPHA+	426	1.6
Bibi, 2010 [69]	2003	Hyderabad	Conv	Red-light district	TPHA+	50	44.0
Raza, 2015 [70]	2014	Rawalpindi	Conv	Clinic	RDT+	NR	20.0
**Somalia:**							
Jama, 1987 [56]	1985-86	Mogadishu	Conv	Community	TPHA+	85	57.6
Jama Ahmed, 1991 [43]	1988-89	Mogadishu	Conv	Community	TPHA+	155	69.0
Burans, 1990 [71]	NR	Mogadishu	Conv	NR	TPHA+	89	28.1
IOM, 2017 [60]	2008	Hargeisa	RDS	Community	RDT+	237	3.4
**Sudan:**							
Sudan NACP, 2012 [72]	2011	Alshamalia	RDS	Community	RDT+	305	1.5
Sudan NACP, 2012 [72]	2011	Blue Nile	RDS	Community	RDT+	279	3.4
Sudan NACP, 2012 [72]	2011	Gadarif	RDS	Community	RDT+	282	3.4
Sudan NACP, 2012 [72]	2011	Gezira	RDS	Community	RDT+	296	5.4
Sudan NACP, 2012 [72]	2011	Kassala	RDS	Community	RDT+	288	4.3
Sudan NACP, 2012 [72]	2011	Khartoum	RDS	Community	RDT+	287	1.7
Sudan NACP, 2012 [72]	2011	North Darfur	RDS	Community	RDT+	303	5.2
Sudan NACP, 2012 [72]	2011	North Kodofan	RDS	Community	RDT+	296	4.1
Sudan NACP, 2012 [72]	2011	Red Sea	RDS	Community	RDT+	293	8.9
Sudan NACP, 2012 [72]	2011	River Nile	RDS	Community	RDT+	291	1.9
Sudan NACP, 2012 [72]	2011	Sinnar	RDS	Community	RDT+	303	5.3
Sudan NACP, 2012 [72]	2011	South Darfur	RDS	Community	RDT+	299	1.8
Sudan NACP, 2012 [72]	2011	West Darfur	RDS	Community	RDT+	284	1.8
Sudan NACP, 2012 [72]	2011	White Nile	RDS	Community	RDT+	288	4.2
MOH, 2016 [61]	2015-16	Juba, South Sudan	RDS	Community	RDT+	832	12.0
**Tunisia**							
Bchir, 1988 [62]	1987	Sousse	Conv	NR	TPHA+	42	38.1
Ayachi, 1997 [63]	1992-94	Tunis	Conv	NR	TPHA+	79	36.7
Znazen, 2010 [73]	2007	Gabes, Sousse, Tunis	Conv	Clinic	TPHA+	183	2.7
**UNCLEAR**							
**Afghanistan:**							
WHO, 2018 [28]	2010	NR	NR	NR	NR	NR	8.7
MENA HIV ESP, 2013 [74]	2012	Kabul	NR	NR	NR	440	5.7
WHO, 2018 [28]	2017	NR	NR	NR	NR	2,457	1.3
**Algeria:**							
WHO, 2018 [28]	2013	Oran	NR	NR	NR	27	7.4
WHO, 2018 [28]	2014	Saida	NR	NR	NR	24	29.2
WHO, 2018 [28]	2016	NR	Conv	VCT	NR	183	14.2
WHO, 2018 [28]	2017	NR	Conv	VCT	NR	81	16.0
**Djibouti:**							
WHO, 2015 [1]	2014	4 urban sites	NR	NR	NR	361	5.0
**Iran:**							
WHO, 2018 [28]	2008	NR	NR	NR	NR	NR	1.6
Moayedi-Nia, 2016 [75]	2012-13	Tehran	RDS	Community	NR	161	0
**Jordan:**							
WHO, 2015 [1]	2008	NR	NR	NR	NR	NR	6.7
**Morocco:**							
Khattabi, 2005 [76]	2004	National	Conv	Prison	NR	332	9.6
Khattabi, 2005 [76]	2004	National	Conv	Clinic	NR	272	12.1
Khattabi, 2005 [76]	2004	Grand Casablanca	Conv	STI clinic	NR	143	9.0
Bennani, 2006 [77]	2005	National	Conv	Prison	NR	102	11.8
Bennani, 2006 [77]	2005	National	Conv	Clinic	NR	143	13.3
WHO, 2018 [28]	2008	NR	NR	NR	NR	NR	16.9
**Pakistan:**							
MENA HIV ESP, 2010 [24]	2007	NR	NR	NR	NR	NR	23.5
**Somalia:**							
WHO, 2018 [28]	2017	Bossaso, Hargeisa, Mogadishu	RDS	Community	NR	860	2.7
**Sudan:**							
WHO, 2018 [28]	2016	National	RDS	Community	NR	4,123	4.1
WHO, 2018 [28]	2017	South Sudan	NR	NR	NR	1,244	14.4
**Yemen:**							
WHO, 2018 [28]	2010	Hodeida	RDS	Community	NR	301	0

Conv – convenience, FTA-ABS – fluoresceent treponemal antibody absorption test, IOM – International Organization for Migration, MENA HIV ESP – MENA HIV/AIDS Epidemiology Synthesis Project database, MOH – Ministry of Health, NACP – National AIDS Control Program, NGO – non-governmental organization, NR – not reported, RDS – respondent-driven sampling, RDT – rapid diagnostic test, RPR – rapid plasma regain, STI – sexually transmitted infection, SyCS – systematic cluster sampling, TPHA – *Treponema pallidum* haemagglutination assay, VCT – voluntary counseling and testing center, VDRL – venereal disease research laboratory

*The table is sorted, for each country, by data collection year(s) then city/province.

†Sample comprised of 77 FSWs and 4 transgender women.

‡Ever infection indicates seropositivity using antibody testing.

Current *C. trachomatis* infection prevalence (n = 14) ranged from 0.7%-72.9%, with a median of 7.7% (IQR = 1.7%-22.4%), while seropositivity prevalence using IgG (n = 5) ranged from 19.8%-100%, with a median of 85.8% (IQR = 46.8%-97.1%; **Table 2**). Two studies reported recent *C. trachomatis* infection (assessed using serological biomarkers) at 29.2% [79] and 95.0% [78].

**Table 2:** Prevalence of *Chlamydia trachomatis*, *Neisseria gonorrhoeae*, and *Trichomonas vaginalis* among FSWs in the Middle East and North Africa*

Country short citation	Year(s) of data collection	City/province	Sampling	Study site	Specimen	Assay type	Tested (n)	Prevalence (%)
**CURRENT INFECTION**								
***Chlamydia trachomatis***								
**Algeria:**								
Kadi, 1989 [78]	NR	NR	Conv	Clinic	Endocervical	IFAT	44	45.5
**Egypt:**								
MOH, 2000 [45]	1999-00	Cairo	Conv	Community	Urine	NAAT	52	7.7
**Iran:**								
Darougar, 1983 [79]	NR	Bandar Abbas, Tehran	Conv	Clinic	Endocervical	Culture	116	6.9
Kazerooni, 2014 [41]	2010-11	Shiraz	RDS	Community	Vaginal	NAAT	278	9.0
Mirzazadeh, 2016 [68]	2015	National	Conv	Clinic, community	Vaginal	NAAT	1337	6.0
**Morocco:**								
MOH, 2008 [48]	2007	Agadir, Rabat Sale, Tanger	Conv	Clinic	Endocervical & urine	NAAT	141	22.7
MOH, 2012 [49]	2011-12	Agadir	RDS	Community	Endocervical	NAAT	368	22.4
**Pakistan:**								
Rehan, 2009 [51]	2004	Karachi	Snowball	Community	Vaginal	NAAT	348	5.2
Rehan, 2009 [51]	2004	Lahore	SyCS	Red-light district	Vaginal	NAAT	283	11.0
Hawkes, 2009 [54]	2007	Abbottabad	RDS	Community	Endocervical	NAAT	107	0.9
Hawkes, 2009 [54]	2007	Rawalpindi	RDS	Community	Endocervical	NAAT	426	1.7
Khan, 2011 [55]	2007	Lahore	RDS	Community	Endocervical	NAAT	730	7.7
**Somalia:**								
IOM, 2017 [60]	2014	Hargeisa	RDS	Community	Urine	NAAT	90	0.7
**Tunisia:**								
Znazen, 2010 [73]	2007	Gabes, Sousse, Tunis	Conv	Clinic	Endocervical	NAAT	188	72.9
***Neisseria gonorrhoeae***								
**Egypt:**								
MOH, 2000 [45]	1999-00	Cairo	Conv	Community	Urine	NAAT	52	7.7
**Iran:**								
Kazerooni, 2014 [41]	2010-11	Shiraz	RDS	Community	Vaginal	Culture	278	1.4
Navadeh, 2012 [42] & WHO, 2011 [80]	2010	Kerman	RDS	Community	NR	NR†	144	0
Nasirian, 2017 [81]	2013-14	Isfahan	Conv	Harm reduction	Endocervical	NAAT	99	9.1
Nasirian, 2017 [81]	2013-14	Isfahan	Conv	Harm reduction	Urine	NAAT	99	0‡
Taghizadeh, 2015 [82]	2014	Sari	Conv	Drop-in center	NR	NR†	117	1.0
Mirzazadeh, 2016 [68]	2015	National	Conv	Clinic, community	Vaginal	NAAT	1337	1.3
**Morocco:**								
MOH, 2008 [48]	2007	Agadir, Rabat Sale, Tanger	Conv	Clinic	Endocervical & urine	NAAT	141	10.6
MENA HIV ESP, 2010 [24]	NR	NR	NR	NR	NR	NR†	NR	3.5
MOH, 2012 [49]	2011-12	Agadir	RDS	Community	Endocervical	NAAT	368	11.7
**Pakistan:**								
Rehan, 2009 [51]	2004	Karachi	Snowball	Community	Vaginal	NAAT	348	9.8
Rehan, 2009 [51]	2004	Lahore	SyCS	Red-light district	Vaginal	NAAT	383	12.3
Hawkes, 2009 [54]	2007	Abbottabad	RDS	Community	Endocervical	NAAT	107	1.9
Hawkes, 2009 [54]	2007	Rawalpindi	RDS	Community	Endocervical	NAAT	426	2.0
Khan, 2011 [55]	2007	Lahore	RDS	Community	Endocervical	NAAT	730	7.5
**Somalia:**								
Burans, 1990 [71]	NR	Mogadishu	Conv	NR	NR	Culture	89	11.2
IOM, 2017 [60]	2014	Hargeisa	RDS	Community	Urine	NAAT	91	0.4
**Tunisia:**								
NACP, 2005 [83]	2005	NR	NR	NR	NR	NR†	NR	12.0-17.0§
Znazen, 2010 [73]	2007	Gabes, Sousse, Tunis	Conv	Clinic	Endocervical	Culture	188	3.7‖
Znazen, 2010 [73]	2007	Gabes, Sousse, Tunis	Conv	Clinic	Endocervical	NAAT	188	11.2
***Trichomonas vaginalis***								
**Egypt:**								
MOH, 2000 [45]	1999-00	Cairo	Conv	Community	Urine	NAAT	52	19.2
**Iran:**								
Vafaei, 2015 [84]	2009-11	Shiraz	Conv	Clinic, drop-in center	Endocervical	Wet mount	85	8.2
Navadeh, 2012 [42] & WHO, 2011 [80]	2010	Kerman	RDS	Community	NR	NR†	144	1.4
Nasirian, 2017 [81]	2013-14	Isfahan	Conv	Harm reduction	Endocervical	NAAT	99	0.0
Nasirian, 2017 [81]	2013-14	Isfahan	Conv	Harm reduction	Urine	NAAT	99	0.0‡
Mirzazadeh, 2016 [68]	2015	National	Conv	Clinic, community	Vaginal	NAAT	1337	11.9
**Morocco:**								
MOH, 2008 [48]	2007	Agadir, Rabat Sale, Tanger	Conv	Clinic	Endocervical & vaginal	Culture	141	14.9
MOH, 2012 [49]	2011-12	Agadir	RDS	Community	Vaginal	NAAT	367	11.8
**Pakistan:**								
Rehan, 2009 [51]	2004	Karachi	Snowball	Community	Vaginal	Culture	386	5.2
Rehan, 2009 [51]	2004	Lahore	SyCS	Red-light district	Vaginal	Culture	384	19.3
Hawkes, 2009 [54]	2007	Abbottabad	RDS	Community	Vaginal	NAAT	107	5.7
Hawkes, 2009 [54]	2007	Rawalpindi	RDS	Community	Vaginal	NAAT	426	4.3
Khan, 2011 [55]	2007	Lahore	RDS	Community	Vaginal	Culture	730	5.1
**RECENT INFECTION**								
***Chlamydia trachomatis***								
**Algeria:**								
Kadi, 1989 [78]	NR	NR	Conv	Clinic	Serum	MIF>1:64¶	44	95.0
**Iran:**								
Darougar, 1983 [79]	NR	Bandar Abbas, Tehran	Conv	Clinic	Serum	MIF-IgM	154	29.2
EVER INFECTION**								
***Chlamydia trachomatis***								
**Algeria:**								
Kadi, 1989 [78]	NR	NR	Conv	Clinic	Serum	MIF-IgG	44	100
**Iran:**								
Darougar, 1983 [79]	NR	Bandar Abbas, Tehran	Conv	Clinic	Serum	MIF-IgG	154	94.2
Kassaian, 2012 [46]	2009-10	Isfahan	Conv	Drop-in center	Serum	ELISA-IgG	91	19.8
**Tunisia**								
Bchir, 1988 [62]	1987	Sousse	Conv	NR	Serum	MIF>1:16	42	73.8
Znazen, 2010 [73]	2007	Gabes, Sousse, Tunis	Conv	Clinic	Serum	MIF-IgG	183	85.8
**UNCLEAR:**								
***Chlamydia trachomatis***								
**Iran:**								
Navadeh, 2012 [42] & WHO, 2011 [80]	2010	Kerman	RDS	Community	NR	NR	144	2.9
**Morocco:**								
MENA HIV ESP, 2010 [24]	NR	NR	NR	NR	NR	NR	NR	19.1

Conv – convenience, ELISA – enzyme-linked immunosorbent assay, IFAT – indirect immunofluorescence antibody test, IgG – immuneglobulin G, IgM - immunoglobulin M, IOM – International Organization for Migration, MENA HIV ESP – MENA HIV/AIDS Epidemiology Synthesis Project database, MIF – micro-immunofluorescence, MOH – Ministry of Health, NAAT – Nucleic acid amplification test, NR – not reported, RDS – respondent-driven sampling, SyCS – systematic cluster sampling, WHO – World Health Organization

*The table is sorted for each country by data collection year(s) then city/province.

†For *Neisseria gonorrhoeae* and *Trichomonas vaginalis* studies, whenever the diagnostic method was not explicitly specified, it was assumed that the diagnostic method assessed current infection.

‡Studies reported in the systematic review, but not included in analyses considering the priority order followed for selecting studies applying the same assay to different biological specimens.

§Range reported based on several studies whose abstracts or full-texts could not be retrieved (mid-point: 14.5%).

‖Studies reported in the systematic review, but not included in analyses as prevalence was also assessed using NAAT.

¶Reported in study as recent infection.

**Ever infection indicates seropositivity using antibody testing.

Current *N. gonorrhoeae* infection prevalence (n = 18) ranged from 0%-14.5%, with a median of 7.6% (IQR = 1.3%-11.1%; **Table 2**). Current *T. vaginalis* infection prevalence (n = 12) ranged from 0%-19.3%, with a median of 7.0% (IQR = 4.5%-14.2%; **Table 2**). HSV-2 seropositivity (using IgG; n = 5) ranged from 4.7%-55.5%, with a median of 20.0% (IQR = 6.4%-39.1%; **Table 3**).

**Table 3:** Prevalence of herpes simplex virus type 2 (HSV-2) immunoglobulin G (IgG) sero-markers among FSWs in the Middle East and North Africa

Country short citation	Year(s) of data collection	City/province	Sampling	Study site	Specimen	Assay type	Tested (n)	Prevalence (%)
**Pakistan:**								
Hawkes, 2009 [54]	2007	Abbottabad	RDS	Community	Serum	ELISA-IgG	107	4.7
Hawkes, 2009 [54]	2007	Rawalpindi	RDS	Community	Serum	ELISA-IgG	426	8.0
**Syria:**								
Ibrahim, 2000 [85]	1995-98	Damascus	Conv	Cheap hotels & prison	Serum	MEIA-IgG	101	22.8
Ibrahim, 2000 [85]	1995-98	Damascus	Conv	Bars	Serum	MEIA-IgG	125	20.0
**Tunisia:**								
Znazen, 2010 [73]	2007	Gabes, Sousse, Tunis	Conv	Clinic	Serum	ELISA-IgG	183	55.5

Conv – convenience, ELISA – enzyme-linked immunosorbent assay. MEIA – micro-enzyme immunoassay. RDS – respondent-driven sampling

### Quality assessment

The summarized and study-specific ROB assessments of prevalence measures are in Tables S5 and S6 in **Online Supplementary Document**, respectively. Briefly, nearly half of studies (44.7%) used probability-based sampling. Most studies (78.7%) indicated explicitly the biological assay used for STI ascertainment. Response rate information was missing in over half of studies (51.8%).

Overall, studies were of reasonable quality. Close to 60% of studies had low ROB on at least two quality domains, and none had high ROB on two or more domains.

### Pooled mean prevalence estimates

**Table 4** shows the results of meta-analyses estimating the pooled mean prevalence of current and/or ever infection for each STI. The mean prevalence of current infection was estimated at 12.7% (95% CI = 8.5%-17.7%) for syphilis, 14.4% (95% CI = 8.2%-22.0%) for *C. trachomatis*, 5.7% (95% CI = 3.5%-8.4%) for *N. gonorrhoeae*, and 7.1% (95% CI = 4.3%-10.5%) for *T. vaginalis*.

**Table 4:** Results of meta-analyses on prevalence studies for *Treponema pallidum* (syphilis), *Chlamydia trachomatis*, *Neisseria gonorrhoeae*, *Trichomonas vaginalis*, and herpes simplex virus type 2 (HSV-2) among FSWs in the Middle East and North Africa

Sexually transmitted infection*	Studies	Samples		Reported prevalence		Pooled mean prevalence		Heterogeneity measures		
N†	Tested	Positive	Median‡ (%)	Range‡ (%)	Estimate (%)	95% CI	Q§ (*P*)	I2‖ (%; 95% CI)	Prediction interval¶ (95%)
**Current infection:**										
*Treponema pallidum* (syphilis)	34	7103	842	10.8	0-62.0	12.7	8.5-17.7	1045.3 (*P* < 0.0001)	96.8 (96.2-97.4)	0.0-48.8
*Chlamydia trachomatis*	16	4608	512	8.4	0.7-76.2	14.4	8.2-22.0	611.4 (*P* < 0.0001)	97.5 (96.9-98.1)	0.0-53.6
*Neisseria gonorrhoeae*	20	5230	301	7.9	0-17.5	5.7	3.5-8.4	248.2 (*P* < 0.0001)	92.3 (89.6-94.4)	0.0-21.6
*Trichomonas vaginalis*	13	4258	397	7.1	0-19.3	7.1	4.3-10.5	164.7 (*P* < 0.0001)	92.7 (89.3-95.0)	0.0-23.7
**Recent infection:**										
*Chlamydia trachomatis*	2**	198	87	62.1	29.2-95.0	–	–	–	–	–
**Ever infection:**††										
*Treponema pallidum* (syphilis)	50	9968	710	7.0	0-92.3	12.8	9.4-16.6	1261.0 (*P* < 0.0001)	96.1 (95.5-96.7)	0.0-45.2
*Chlamydia trachomatis*	6	514	395	84.7	19.8-100	80.3	53.2-97.6	213.0 (*P* < 0.0001)	97.7 (96.4-98.5)	0.0-100.0
Herpes simplex virus type 2 IgG	8	942	188	20.3	4.7-59.7	23.7	10.2-40.4	185.0 (*P* < 0.0001)	96.2 (94.3-97.5)	0.0-84.9
**Unclear:**										
*Treponema pallidum* (syphilis)	22	12 698	771	8.9	0-29.2	7.7	5.1-10.7	591.3 (*P* < 0.0001)	96.4 (95.5-97.2)	0.0-25.7
*Chlamydia trachomatis*	2**	293	32	11.0	2.9-19.1	–	–	–	–	–

CI – confidence interval, FSWs – female sex workers, IgG – immunoglobulin G, P – *P*-value

*The same population may have contributed different measures for both current infection and ever (seropositivity using antibody testing) infection.

†Missing sample sizes for measures (or their strata) were imputed using the median sample size calculated from studies with available information (only two stratified measures for *Neisseria gonorrhoeae*, one stratified measure for *Chlamydia trachomatis*, one stratified measure for current syphilis infection, 5 stratified measures of unclear syphilis infection, had their sample size imputed, that is 5% of all data).

‡Medians and ranges were calculated based on the stratified prevalence measures.

§Q – the Cochran’s Q statistic is a measure assessing the existence of heterogeneity in effect size (here, prevalence) across studies.

‖I2 – a measure assessing the magnitude of between-study variation that is due to differences in effect size (here, prevalence) across studies rather than chance.

¶Prediction interval: a measure estimating the 95% interval of the distribution of true effect sizes (here, prevalence measures).

**Meta-analyses were performed if at least three studies were available.

††Ever infection indicates seropositivity using antibody testing.

The mean prevalence of ever infection was estimated at 12.8% (95% CI = 9.4%-16.6%) for syphilis, 80.3% (95% CI = 53.2%-97.6%) for *C. trachomatis*, and 23.7% (95% CI = 10.2%-40.4%) for HSV-2 IgG.

There was strong evidence for heterogeneity in effect size (here, prevalence). *P* for Cochran’s Q statistic was always <0.0001. I^2^ was >90% in all meta-analyses, indicating that most variability is due to true differences in effect size across studies, rather than being due to chance. Prediction intervals were also wide affirming high heterogeneity.

Additional meta-analyses at the subregional level indicated the mean prevalence of current syphilis infection at 3.0% (95% CI = 0.9%-9.2%) in Eastern MENA, 17.6% (95% CI = 14.2%-21.3%) in North Africa, and 27.8% (95% CI = 15.2%-42.4%) in the Horn of Africa (Table S7 in **Online Supplementary Document**). There was also a tendency for a decline in current infection prevalence post-2010 (Table S8 and Figure S1A in **Online Supplementary Document**). For the rest of the STIs, the number of studies was small and the CIs were wide and overlapping to warrant conclusive statement about the temporal trend (Table S8 in **Online Supplementary Document**).

### Predictors of variability in syphilis infection

Country/subregion, year of data collection, diagnostic method, sample size, sampling methodology, and response rate were associated with higher odds of syphilis infection in the univariable meta-regression analyses. These were, therefore, included in the multivariable model (**Table 5**). About a third of the variability was explained by each of year of data collection and subregion (adjusted R-squared: 34.6% and 31.5%, respectively). Meanwhile, no evidence for an association with infection type (current infection; ever infection), or STI ascertainment (biological assay explicitly indicated; otherwise) was found.

**Table 5:** Results of meta-regression analyses to identify associations and sources of between-study heterogeneity in syphilis prevalence in the Middle East and North Africa (MENA)

Factors		Studies	Samples	Univariable analyses				Multivariable analysis*		
Total N	Total n	OR† (95% CI)	*P*	*P*‡ of LR test	Variance explained R2 (%)	AOR† (95% CI)	*P*	*P*§ of LR test
**Country/subregion:‖**										
Eastern MENA	Afghanistan, Iran, Pakistan	28	10 865	1.00		<0.001	31.52	1.00		<0.001
Egypt, Jordan, Yemen	Egypt, Jordan, Yemen	4	881	0.89 (0.15-5.10)	0.893			0.66 (0.13-3.28)	0.609	
North Africa	Algeria, Morocco, Sudan, Tunisia	48	12 394	5.34 (2.45-11.61)	<0.001			5.01 (2.37-10.61)	<0.001	
Horn of Africa	Djibouti, Somalia, South Sudan	26	5629	21.63 (8.89-52.69)	<0.001			6.40 (2.45-16.69)	<0.001	
**Year of data collection**¶		106	29 769	0.88 (0.85-0.91)	<0.001	<0.001	34.61	0.93 (0.88-0.98)	0.005	0.005
**Infection type**	Current	34	7103	1.00		0.515	0.00	–	–	–
	Ever**	50	9968	1.25 (0.52-3.00)	0.622			–	–	–
	Unclear	22	12 698	0.69 (0.23-2.04)	0.501			–	–	–
**Diagnostic method**	RPR/VDRL & TPHA/FTA-ABS/RDT	29	6095	1.00		<0.001	22.44	1.00		0.444
	RPR/VDRL	4	488	0.09 (0.01-0.61)	0.013			0.76 (0.15-4.00)	0.746	
	TPHA	28	1781	2.17 (0.86-5.45)	0.099			1.29 (0.54-3.07)	0.558	
	RDT	23	8707	0.17 (0.06-0.45)	<0.001			0.46 (0.18-1.18)	0.104	
	Not specified	22	12 698	0.43 (0.16-1.16)	0.094			0.75 (0.24-2.33)	0.614	
**STI ascertainment**	Biological assay not reported	23	13 066	1.00		0.284	0.15	–	–	–
	Biological assay explicitly indicated	83	16 703	1.66 (0.65-4.20)	0.284			–	–	–
**Sample size**	<100 participants	42	1960	1.00		<0.001	20.02††	1.00		
	≥100 participants	64	27 809	0.16 (0.08-0.32)	<0.001			1.60 (0.62-4.15)	0.329	0.329
**Sampling methodology**	Non-probability/unclear sampling	66	12 555	1.00		<0.001	18.73‡‡	1.00		
	Probability-based sampling	40	17 214	0.16 (0.08-0.34)	<0.001			0.63 (0.25-1.63)	0.339	0.339
**Response rate**	<60%/unclear	69	18 400	1.00		<0.001	10.23§§	1.00		
	≥60%	37	11 369	0.25 (0.12-0.54)	0.001			0.73 (0.29-1.84)	0.495	0.495

AOR – adjusted odds ratio, CI – confidence interval, FTA-ABS – fluorescent treponemal antibody absorption test, LR – likelihood ratio, OR – odds ratio, P – *P*-value, RDT – rapid diagnostic test, RPR – rapid plasma regain, STI – sexually transmitted infection TPHA – *Treponema pallidum* haemagglutination assay. VDRL – venereal disease research laboratory

*Adjusted R^2^ in the multivariable model: 48.46%.

†An increment of 0.1 was added to number of events when generating log odds of syphilis infection. This is because 8 stratified measures had zero events.

‡Factors with *P* ≤ 0.1 were eligible for inclusion in the multivariable analysis.

§Factors with *P* < 0.05 in the multivariable model were considered as significant predictors.

‖Countries were grouped based on geography and similarity in prevalence levels.

¶Missing values for year of data collection (only one stratified measure) were imputed using data for year of publication adjusted by the median difference between year of publication and median year of data collection for studies with complete information.

**Ever infection indicates seropositivity using antibody testing.

††The high R^2^ was investigated and found to be due to confounding with year of data collection. Most studies with sample size ≥100 were conducted in recent years.

‡‡The high R^2^ was investigated and found to be due to confounding with country and year of data collection. Studies with non-probability sampling were mostly from the Horn of Africa. These studies tended also to be conducted in earlier years.

§§The high R^2^ was investigated and found to be due to confounding with year of data collection. Most studies with response rate ≥60% were conducted in recent years.

The multivariable analysis showed strong evidence for subregional differences, with Horn of Africa and North Africa showing, respectively, 6-fold (adjusted odds ratio (AOR): 6.4; 95% CI = 2.5-16.7) and 5-fold (AOR = 5.0; 95% CI = 2.5-10.6), higher odds of syphilis infection than Eastern MENA.

There was also strong evidence for a temporal trend of decreasing odds of infection at 7% per year (AOR = 0.93; 95% CI = 0.88-0.98; linearity dictated by data (Figure S1 in **Online Supplementary Document**) over the last three decades. Although this trend was noted in all subregions, individual subregion meta-regressions were not always powered to detect statistical significance (not shown).

No evidence for an association with diagnostic method, sample size, sampling methodology, and response rate was identified in the multivariable model. The multivariable model explained 48.5% of variation in syphilis prevalence.

## DISCUSSION

We provided, to our knowledge, the first detailed assessment of the epidemiology of key STIs in FSWs in MENA, a neglected key population. Our findings indicated substantial STI prevalence, several folds higher than that among the general population [2,13,24,86]. These findings suggest a major role for CHSNs in driving STI transmission in MENA. We further found large heterogeneity in syphilis infection levels by subregion within MENA, as well as a trend of decreasing odds of infection by ~ 7% per year – less than the 17% [86] annual decline needed to achieve the target of 90% reduction in syphilis incidence by 2030 [6].

Despite the significant infection burden, STI surveillance and response in MENA continue to be rudimentary [21], and far below the coverage targets of WHO Global Health Sector Strategy for STIs [6]. Infected individuals are often identified through routine case notifications with surveillance/testing being largely limited to HIV [21,24,87], and sexual health programs, where they exist, cater to general population women rather than women at high risk [24].

Although our expansive search identified considerable evidence at the regional-level, including data that will appear in the scientific literature for the first time, evidence varied by country. Over half of countries had no data on any of the STIs in this key population, less than a third had data on *C. trachomatis*, *N. gonorrhoeae*, or *T. vaginalis*, and only three countries had data on HSV-2 IgG (**Table 1**, **Table 2** and **Table 3**). This outcome is of concern, given the considerable, yet preventable, STI infection burden among FSWs in the region (**Table 4**), and the major “core group” role that CHSNs play in STI transmission in any population [10]. Indeed, while the population proportion of FSWs (proportion of FSWs out of the total women population) varies across countries and may seem relatively small [18,88], the size of CHSNs is large suggesting a considerable number of women and men at risk of STI-related morbidity, either through engagement in high sexual risk behavior, or through onward infection transmission [89].

Availability of STI data stands in contrast to HIV data, for which the volume of evidence among FSWs was several fold higher and encompassed most countries [18]. Attending to WHO Global Health Sector Strategy on STIs [6] necessitates a major expansion of STI research and surveillance, as has been done for HIV [17,87,90]. Regrettably, integrated bio-behavioural surveillance surveys (IBBSS) among key populations continue to be focused on HIV, rarely incorporating STIs [91,92]. This presents an important, yet lost, opportunity for monitoring STI levels and trends in key populations, informing programming efforts, gaining an in-depth understanding of sexual networks’ structure, and advancing STI research in this region [13,91,93].

Subregion and time explained most variation in syphilis prevalence—each explained over a third of the variation, and both (remarkably) explained ~ 50% of the variation (**Table 5**). The strong subregional differences, with Horn of Africa showing the highest prevalence, followed by North Africa, and then Eastern MENA **(****Table 5** and Table S7 in **Online Supplementary Document**), appear to reflect variability in the risk environment, such as differences in structure of sexual networks [24], condom use [18], and access to care [24]. The same pattern has been seen in HIV epidemiology among FSWs [18].

There was strong evidence for a time trend of decreasing odds of infection at ~ 7% per year (**Table 5**, and Table S8 and Figure S1 in the OSD), consistent with, but smaller than, the decline reported for the general population in MENA in a recent global analysis [86], and the declines reported for the general populations in other regions [86]. Different factors may have contributed to this trend including safer sex following the HIV epidemic [94], increased condom use to prevent unwanted pregnancy [18], and HIV-related mortality which may have disproportionally affected populations at higher risk of STIs [95]. This may have been also a consequence of a shorter duration of active syphilis infection in FSWs or their sex partners [96,97], possibly because of improvements in syphilis diagnostics and treatment, or because of widespread use of antibiotics (including for non-STI infections, which sometimes may cure concurrent syphilis) [86].

This being said, recent surveillance data seems also to suggest an increase in syphilis incidence and/or prevalence in other sexual networks or in specific settings, such as among MSM [98-100], and even among reproductive-age women in few countries where congenital syphilis appears to be rising [101,102]. Contributors to these trends may include behavioral factors, such as more sexual partners and unprotected sex among MSM, as well as contextual factors, and possibly even biological factors [99,100,102-104].

Prevalence measures for syphilis and for *C. trachomatis* in FSWs in MENA were comparable to global levels [22,23], but prevalence measures for *N. gonorrhoeae* and *T. vaginalis* leaned towards the lower end of the global range [22,23]. Even though the risk environment among FSWs in MENA seems less conducive to STI transmission, as compared to other regions [18], STI prevalence levels are substantial, perhaps affected by poor access to health care and prevention interventions [21,24,105], as well as absence of enabling environments for this vulnerable population, in a context of criminality [106,107] and stigma [108-110].

While interventions aiming at promoting safer sex, such as condom use, and STI etiological diagnosis and treatment, in high risk populations are widely accepted and advocated for [6,111-114], STI syndromic case management and presumptive treatment have been increasingly subject to criticism amid growing concerns about their role in promoting pathogens’ antimicrobial resistance (AMR) [111,115-119]. Indeed, substantial AMR prevalence and multiple drug resistant strains have been found in gonococcal isolates from FSWs in sub-Saharan Africa [120,121] and elsewhere [122]. This suggests that despite the effectiveness of targeted STI treatment services in reducing STI incidence and prevalence, their appropriateness and sustainable implementation will need to be informed by surveillance and monitoring, notably for AMR, and thus may vary across settings [111,122]. This further supports WHO efforts towards building a global business case for accelerating development of STI vaccines as a fundamental solution to STI drug resistance [123-125].

This study is limited by the quantity and quality of available data. STI prevalence among FSWs remains unknown in over half of countries. While there was considerable evidence for syphilis, less evidence was found for *C. trachomatis*, *N. gonorrhoeae*, *T. vaginalis*, and HSV-2, limiting our ability to conduct advanced meta-analytics—meta-regressions were carried out only for syphilis. Though, for syphilis prevalence, the differences between current vs ever (seropositivity using antibody testing) infection, as well as the differences between diagnostics, were consistent with the findings of a large global analysis for the general population [86], the confidence intervals were wide owing to the smaller number of studies (**Table 5**). Several measures were based on routine data reporting, and did not include sufficient documentation of study methodology. There was also a wide array of diagnostics used for STI ascertainment, which may have affected observed prevalence.

Available studies may not be representative of the wider population of FSWs, or could be subject to biases, such as selection bias or detection bias. Of note, however, that there was no evidence that any of the assessed study-specific quality domains (Tables S5-S6 in **Online Supplementary Document**), including sampling methodology, response rate, and explicit indication of the assay used for infection ascertainment, had an effect on prevalence in the multivariable meta-regression (**Table 5**). Despite limitations, our study provided a detailed synthesis of STI epidemiology in FSWs in MENA, in a background of lack of evidence for this region [22,23]. A significant volume of published and unpublished data was identified and analyzed, and for the first time.

In conclusion, STI levels among FSWs are considerable, supporting a key role for CHSNs in STI transmission dynamics in MENA, and highlighting the public health needs of this neglected and vulnerable population. Despite the progress in our epidemiological understanding, major gaps persist, with no evidence being available for over half of MENA countries. With the limited STI surveillance [24,126], and the focus of programmatic response on case management and syndromic approach, rather than being evidence-informed and grounded on etiological studies [24,126], there is a critical need to expand STI surveillance and the broader STI research agenda. STI testing should be part of IBBSS studies, as well as part of voluntary counseling and testing services for HIV [91,93]. Interventions should factor research findings to ensure adequate and efficient resource allocation. Without such expansion of STI efforts, it will not be possible to monitor infection trends, or to inform a public health response that attends to the WHO Global Health Sector Strategy on STIs [6].

## Additional material

Online Supplementary Document
