# Supplementary material for: Epidemiology of Treponema pallidum, Chlamydia trachomatis, Neisseria gonorrhoeae, Trichomonas vaginalis, and herpes simplex virus type 2 among female sex workers in the Middle East and North Africa: systematic review and meta-analytics
Source: J Glob Health. 2019 Jul 21;9(2):020408. doi: 10.7189/jogh.09.020408 (PMC6642815; doi:10.7189/jogh.09.020408)
Supplement: Online Supplementary Document [file jogh-09-020408-s001.pdf]

J Glob Health 2019; 9: 020408

Online Supplementary Document

Table S1. Preferred Reporting Items for Systematic Reviews and Meta-analyses (PRISMA) checklist [1]

Section/topic	#	Checklist item	Reported in main text
Title	1	Identify the report as a systematic review, meta-analysis, or both.	p. 1
Structured summary	2	Provide a structured summary including, as applicable: background; objectives; data sources; study eligibility criteria, participants, and interventions; study appraisal and synthesis methods; results; limitations; conclusions and implications of key findings; systematic review registration number.	p. 2-3
Rationale	3	Describe the rationale for the review in the context of what is already known.	p. 4-5
Objectives	4	Provide an explicit statement of questions being addressed with reference to participants, interventions, comparisons, outcomes, and study design (PICOS).	p. 5
Protocol and registration	5	Indicate if a review protocol exists, if and where it can be accessed (e.g., Web address), and, if available, provide registration information including registration number.	NA
Eligibility criteria	6	Specify study characteristics (e.g., PICOS, length of follow-up) and report characteristics (e.g., years considered, language, publication status) used as criteria for eligibility, giving rationale.	p. 6-7
Information sources	7	Describe all information sources (e.g., databases with dates of coverage, contact with study authors to identify additional studies) in the search and date last searched.	p. 6
Search	8	Present full electronic search strategy for at least one database, including any limits used, such that it could be repeated.	Box S1 in the OSD
Study selection	9	State the process for selecting studies (i.e., screening, eligibility, included in systematic review, and, if applicable, included in the meta-analysis).	p. 6-7
Data collection process	10	Describe method of data extraction from reports (e.g., piloted forms, independently, in duplicate) and any processes for obtaining and confirming data from investigators.	p. 7
Data items	11	List and define all variables for which data were sought (e.g., PICOS, funding sources) and any assumptions and simplifications made.	p. 7 and Box S2 in the OSD
Risk of bias in individual studies	12	Describe methods used for assessing risk of bias of individual studies (including specification of whether this was done at the study or outcome level), and how this information is to be used in any data synthesis.	p. 8 and Table S3 in the OSD
Summary measures	13	State the principal summary measures (e.g., risk ratio, difference in means).	p. 8-9
Synthesis of results	14	Describe the methods of handling data and combining results of studies, if done, including measures of consistency (e.g., I^2) for each meta-analysis.	p. 8-9
Risk of bias across studies	15	Specify any assessment of risk of bias that may affect the cumulative evidence (e.g., publication bias, selective reporting within studies).	p. 8
Additional analyses	16	Describe methods of additional analyses (e.g., sensitivity or subgroup analyses, meta-regression), if done, indicating which were pre-specified.	p.8- 9 and Table S4 in the OSD
Study selection	17	Give numbers of studies screened, assessed for eligibility, and included in the review, with reasons for exclusions at each stage, ideally with a flow diagram.	p. 10 and Figure 1

Section/topic	#	Checklist item	Reported in main text
Study characteristics	18	For each study, present characteristics for which data were extracted (e.g., study size, PICOS, follow-up period) and provide the citations.	p. 11 and Tables 1-3
Risk of bias within studies	19	Present data on risk of bias of each study and, if available, any outcome-level assessment (see Item 12).	p. 11- and Tables S5-S6 in the OSD
Results of individual studies	20	For all outcomes considered (benefits or harms), present, for each study: (a) simple summary data for each intervention group and (b) effect estimates and confidence intervals, ideally with a forest plot.	p. 12 and Table 4
Synthesis of results	21	Present results of each meta-analysis done, including confidence intervals and measures of consistency.	p. 12 and Table 4
Risk of bias across studies	22	Present results of any assessment of risk of bias across studies (see Item 15).	p. 11-12 and Tables S5-S6 in the OSD
Additional analysis	23	Give results of additional analyses, if done (e.g., sensitivity or subgroup analyses, meta-regression [see Item 16]).	p. 12-13 and Table 5
		**DISCUSSION**	
Summary of evidence	24	Summarize the main findings including the strength of evidence for each main outcome; consider their relevance to key groups (e.g., health care providers, users, and policy makers).	p. 14-17
Limitations	25	Discuss limitations at study and outcome level (e.g., risk of bias), and at review level (e.g., incomplete retrieval of identified research, reporting bias).	p. 17-18
Conclusions	26	Provide a general interpretation of the results in the context of other evidence, and implications for future research.	p. 18
Funding	27	Describe sources of funding for the systematic review and other support (e.g., supply of data); role of funders for the systematic review.	p. 19

NA, not applicable. P, page(s). OSD, Online Supplementary Document.

Box S1. Search criteria for the systematic review of *Treponema pallidum* (syphilis), *Chlamydia trachomatis*, *Neisseria gonorrhoeae*, *Trichomonas vaginalis*, and herpes simplex virus type 2 (HSV-2) among FSWs in the Middle East and North Africa (MENA)

PubMed (September 04, 2018)

Sexually transmitted infections

"Sexually transmitted diseases"[Mesh] OR "Reproductive tract infections"[Mesh] OR "Genital diseases, female"[Mesh] OR "Chlamydia trachomatis"[Mesh] OR "Chlamydia"[Mesh] OR "Pelvic inflammatory disease"[Mesh] OR Chlamydia trachomatis[Text] OR Chlamydia[Text] OR Trachomatis[Text] OR Pelvic inflammatory disease[Text] OR Chlamydial[Text] OR Chlamydial infection[Text] OR Chlamydia infection[Text] OR "Neisseria gonorrhoeae"[Mesh] OR "Gonorrhea"[Mesh] OR Neisseria gonorrhoeae[Text] OR Gonorrhoeae[Text] OR Gonorrhea[Text] OR Gonococcus[Text] OR Gonococci[Text] OR Gonococcal[Text] OR Gonococcal infection[Text] OR "Syphilis"[Mesh] OR "Treponema pallidum"[Mesh] OR Syphilis[Text] OR Treponema pallidum[Text] OR Great Pox[Text] OR "Simplexvirus"[Mesh] OR "Herpes Simplex"[Mesh] OR Simplex virus[Text] OR "Herpes Genitalis"[Mesh] OR HSV type-2[Text] OR HSV type 2[Text] OR HSV2[Text] OR HSV-2[Text] OR HSV[Text] OR Human herpes virus[Text] OR Herpes simplex virus type 2[Text] OR Herpes simplex virus type-2[Text] OR Herpes simplex virus 2[Text] OR Herpes simplex virus-2[Text] OR Herpes simplex type 2[Text] OR Herpes simplex type-2[Text] OR Herpes simplex 2[Text] OR Herpes simplex-2[Text] OR Herpesvirus type 2[Text] OR Herpesvirus type-2[Text] OR Herpesvirus 2[Text] OR Herpesvirus-2[Text] OR Herpes virus type 2[Text] OR Herpes virus type-2[Text] OR Herpes virus[Text] OR Herpes virus-2[Text] OR genital herpes[Text] OR Herpes genitalis[Text] OR Stomatitis herpetic[Text] OR Herpes labialis[Text] OR HSV type-1[Text] OR HSV type 1[Text] OR HSV1[Text] OR HSV-1[Text] OR HSV 1[Text] OR Herpes simplex virus type 1[Text] OR Herpes simplex virus type-1[Text] OR Herpes simplex virus 1[Text] OR Herpes simplex virus-1[Text] OR Herpes simplex type 1[Text] OR Herpes simplex type-1[Text] OR Herpes simplex 1[Text] OR Herpes simplex-1[Text] OR Herpesvirus type 1[Text] OR Herpesvirus type-1[Text] OR Herpesvirus 1[Text] OR Herpesvirus-1[Text] OR Herpes virus type 1[Text] OR Herpes virus type-1[Text] OR Herpes virus 1[Text] OR Herpes virus-1[Text] OR Sexually transmitted[Text] OR Venereal[Text] or STI[Text] or STD[Text] or genital[Text] or infection[Text] or infections[Text] or infected[Text]

Sex work

"Extramarital Relations"[Mesh] OR "Sex Work*"[Mesh] OR "Sex/analysis"[Mesh] OR "Sex/statistics and numerical data"[Mesh] OR "Sexual partners"[Mesh] OR "Sex Trafficking/epidemiology"[Mesh] OR "Sex Trafficking/statistics and numerical data"[Mesh] OR Sex work*[Text] OR Sexual work*[Text] OR Sexwork*[Text] OR Sex-work*[Text] OR Sexual partner*[Text] OR Sex partner*[Text] OR Sexual contact*[Text] OR FSW[Text] OR FSWs[Text] OR CSW[Text] OR CSWs[Text] OR SW[Text] OR SWs[Text] OR TSW[Text] OR TSWs[Text] OR TS[Text] OR Travailleuse* sexe[Text] OR Travailleuse* sex[Text] OR Bar girl*[Text] OR Callgirl*[Text] OR Call girl*[Text] OR Escort*[Text] OR Masseuse*[Text] OR Hostess*[Text] OR ((Premarital[Text] OR Pre-marital[Text] OR Pre marital[Text] OR Extramarital[Text] OR Extra-marital[Text] OR Extra marital[Text] OR Illicit[Text] OR Illegal[Text]) AND (Sex[Text] OR Sexual[Text] OR Relation*[Text])) OR Outside marriage[Text] OR Out of marriage[Text] OR "Illegal social behavior"[Text] OR "Illegal social behaviour"[Text] OR Adultery[Text] OR Prostitut*[Text] OR Promiscu*[Text] OR Female entertain*[Text] OR Sex entertain*[Text] OR Sexual* entertain*[Text] OR Entertainment work*[Text] OR Sex industr*[Text] OR Sex establishment*[Text] OR Brothel*[Text] OR Red light[Text] OR Red-light[Text] OR Red district*[Text] OR Nightclub*[Text] OR Pimp[Text] OR ((Intergenerational[Text] OR Cross-generation*[Text] OR Cross-generational[Text] OR Recreational[Text] OR Commercial[Text] OR Transaction*[Text] OR Casual[Text] OR Group[Text] OR Informal[Text] OR Street[Text] OR Migrant*[Text] OR Survival[Text] OR Occupational[Text] OR Tourism[Text]) AND (Sex[Text] OR Sexual*[Text])) OR Sex seeking[Text] OR Sex-seeking[Text] OR Solicit*[Text] OR ((Provision*[Text] OR Provider*[Text] OR Provid*[Text] OR Sell*[Text] OR Sold[Text] OR Exchang*[Text] OR Trad*[Text] OR Favor*[Text] OR Consum*[Text] OR Commodi*[Text] OR Paid[Text] OR Paying[Text] OR Pay[Text] OR Payer*[Text] OR Buying[Text] OR Buy[Text] OR Buyer*[Text] OR Charg*[Text] OR Engag*[Text] OR Service*[Text] OR Money[Text] OR Cash[Text] OR Drug*[Text] OR Goods[Text] OR Gift*[Text]) AND (Sex[Text] OR Sexual*[Text])) OR Hidden population*[Text] OR Hard to reach population*[Text] OR Hard-to-reach population*[Text] OR Core group*[Text] OR Core risk group*[Text] OR Vulnerable women[Text] OR Vulnerable population*[Text] OR Vulnerable female*[Text] OR Most-at-risk population*[Text] OR Most at risk population*[Text] OR High risk population*[Text] OR High-risk population*[Text] OR Population* at high risk[Text] OR Population* at high-

risk[Text] OR ((Traffick*[Text] OR Slave*[Text] OR Coerc*[Text] OR Abduct*[Text] OR Exploit*[Text] OR Abuse*[Text] OR Violence[Text]) AND (Sex[Text] OR Sexual*[Text]))

MENA

"Middle East"[Mesh] OR "Islam"[Mesh] OR "Arabs"[Mesh] OR "Arab World"[Mesh] OR "Africa, Northern"[Mesh] OR "Sudan"[Mesh] OR "Somalia"[Mesh] OR "Djibouti"[Mesh] OR "Pakistan"[Mesh] OR "South Sudan"[Mesh] OR "Middle East*"[Text] OR "Middle-East"[Text] OR "North Africa*"[Text] OR "North-Africa"[Text] OR "EMRO"[Text] OR "Eastern Mediterranean"[Text] OR "Arab*"[Text] OR "Arab World"[Text] OR "Islam*"[Text] OR "Afghanistan"[Text] OR "Afghan*"[Text] OR "Algeria*"[Text] OR "Bahrain*"[Text] OR "Djibouti"[Text] OR "Egypt*"[Text] OR "Jordan*"[Text] OR "Kuwait*"[Text] OR "Lebanon"[Text] OR "Leban*"[Text] OR "Libya*"[Text] OR "Iran*"[Text] OR "Iraq*"[Text] OR "Morocco"[Text] OR "Moroccan*"[Text] OR "Oman*"[Text] OR "Pakistan*"[Text] OR "Qatar*"[Text] OR "Saudi*"[Text] OR "Somalia"[Text] OR "Somal*"[Text] OR "Sudan*"[Text] OR "Syria*"[Text] OR "Tunisia*"[Text] OR "United Arab Emirates"[Text] OR "Emirat*"[Text] OR "West Bank"[Text] OR "Ghaza*"[Text] OR "Gaza*"[Text] OR "Palestine"[Text] OR "Palestinian*"[Text] OR "Yemen*"[Text] OR "UAE"[Text] OR "KSA"[Text]

Women

"Female/analysis"[Mesh] OR "Female/statistics and numerical data"[Mesh] OR "Women/epidemiology"[Mesh] OR "Women/statistics and numerical data"[Mesh] OR Women[Text] OR Girl*[Text] OR Female*[Text]

FINAL PUBMED SEARCH
"Sexually transmitted infections" AND "Sex work" AND "MENA" AND "Women"

Embase (September 04, 2018)

Sexually transmitted infections

exp sexually transmitted disease/ or exp chlamydia/ or exp chlamydia trachomatis/ or exp pelvic inflammatory disease/ or exp genital tract infection/ or exp genital tract inflammation/ or chlamydia.mp. or chlamydia trachomatis.mp. or trachomatis.mp. or chlamydial.mp. or chlamydial infection.mp. or chlamydia infection.mp. or pelvic inflammatory disease.mp. or exp gonorrhea / or exp neisseria gonorrhoeae / or gonorrhea.mp. or neisseria gonorrhoeae.mp. or gonorrhoeae.mp. or gonococcus.mp. or gonococci.mp. or gonococcal.mp. or gonococcal infection.mp. or exp syphilis/ or exp treponema pallidum/ or syphilis.mp. or great pox.mp. or treponema pallidum.mp. or exp herpes simplex virus/ or exp herpes simplex/ or exp herpes simplex virus 1/ or exp simplexvirus/ or exp herpesvirus/ or exp herpesviridae/ or exp herpes simplex virus 2/ or (herpes simplex or herpes simplex virus or HSV type-1 or HSV type 1 or HSV1 or HSV-1 or HSV 1 or human herpes virus or herpes simplex virus type 1 or Herpes simplex virus type-1 or herpes simplex virus 1 or herpes simplex virus-1 or herpes simplex type 1 or herpes simplex type-1 or herpes simplex 1 or herpes simplex-1 or Herpesvirus type 1 or Herpesvirus type-1 or Herpesvirus 1 or Herpesvirus-1 or Herpes virus type 1 or Herpes virus type-1 or Herpes virus 1 or Herpes virus-1 or genital herpes or herpes genitalis or herpes labialis or herpetic stomatitis or HSV type-2 or HSV type 2 or HSV2 or HSV-2 or HSV 2 or herpes simplex virus type 2 or herpes simplex virus type-2 or herpes simplex virus 2 or herpes simplex virus-2 or herpes simplex type 2 or herpes simplex type-2 or herpes simplex 2 or herpes simplex-2 or herpesvirus type 2 or herpesvirus type-2 or herpesvirus 2 or herpesvirus-2 or herpes virus type 2 or herpes virus type-2 or herpes virus 2 or Herpes virus-2 or sexually transmitted or venereal or STI or STD or genital or infection or infections or infected).mp.

Sex work

exp prostitution/ or exp casual sex/ or exp transactional sex/ or exp group sex/ or exp sex tourism/ or exp sexual promiscuity/ or exp extramarital sex/ or exp premarital sex/ or exp sexual relation/ or exp sexual partners/ or ((exp sex trafficking/ or exp sexual exploitation/ or exp sexual coercion/) NOT Child) or (sex* work* or sexwork* or sex-work* or sex partner* or sexual partner* or sexual contact* or premarital sex or premarital sexual or premarital relation* or pre-marital sex or pre-marital sexual or pre-marital relation* or pre marital sex or pre marital sexual or pre marital relation* or extramarital sex or extramarital sexual or extramarital relation* or extra-marital sex or extra-marital sexual or extra-marital relation* or extra marital sex or extra marital sexual or extra marital relation* or illicit sex or illicit sexual or illicit relation* or illegal sex or illegal sexual or illegal relation* or (out* ADJ1 marriage) or illegal social behavio?r or adultery or prostitut* or promiscu* or FSW or FSWs or CSW or CSWs or SW or SWs or TSW or TSWs or TS or (women ADJ4 sex*) or (Travailleuse* ADJ1 sex*) or bar girl* or call girl* or callgirl* or escort* or masseuse* or hostess* or female entertain* or sex

entertain* or sexual entertain* or entertainment work* or sex industr* or sex establishment* or brothel* or red light or red-light or (red ADJ1 district*) or nightclub* or pimp or recreation* sex* or intergenerational sex* or cross-generation sex* or cross-generational sex* or commercial sex* or transactional sex* or sex* transaction* or casual sex* or informal sex* or group sex* or street sex* or (migra* ADJ4 sex*) or (sex* ADJ4 migra*) or survival sex* or occupational sex* or sex* tourism or sex seeking or sex-seeking or solicit* or (consum* ADJ4 sex*) or (sex* ADJ 4 consumer) or (sex* ADJ4 consumers) or (sex* ADJ4 provi*) or (provi* ADJ4 sex*) or (sell* ADJ4 sex*) or (sex* ADJ4 sell*) or sold sex* or (exchang* ADJ4 sex*) or (sex* ADJ4 exchange) or (trading ADJ4 sex*) or (trade* ADJ4 sex*) or sex* trade or sex* favor* or (commodi* ADJ4 sex*) or (sex* ADJ4 commodi*) or (paid ADJ4 sex*) or (pay* ADJ4 sex*) or (sex* ADJ4 pay*) or (buy* ADJ4 sex*) or (sex* ADJ4 buy*) or (charg* ADJ4 sex*) or (sex* ADJ4 charg*) or (engag* ADJ4 sex*) or (sex* ADJ4 engage*) or (sex* ADJ4 service*) or (service* ADJ4 sex*) or (money ADJ4 sex*) or (sex* ADJ4 money) or (cash ADJ4 sex*) or (sex* ADJ4 cash) or (sex* ADJ4 drug*) or (drug* ADJ4 sex*) or (sex* ADJ4 goods) or (goods ADJ4 sex*) or (sex* ADJ4 gift*) or (gift* ADJ4 sex*) or hidden population* or hard to reach population* or hard-to-reach population* or (core ADJ1 group*) or vulnerable women or vulnerable female*).mp. or ((vulnerable population* or most-at-risk population* or most at risk population* or high risk population* or high-risk population* or population* at high risk or population* at high-risk).mp. AND (sex* or infection* or STI or STIs or STD or STDs or human immunodeficiency virus or HIV* or AIDS* or acquired immune deficiency syndrome or acquired immunodeficiency syndrome).mp.) or ((sex trafficking or sexual trafficking or (traffick* ADJ4 sex*) or sex* slave* or sex* coerc* or sex* abduct* or sex* exploit* or sex* abuse* or sex* violence) NOT Child).mp. or ((women ADJ4 traffick*) or (girls ADJ4 traffick*) or (female* ADJ4 traffick*) or (traffick* ADJ4 women) or (traffick* ADJ4 girls) or (traffick* ADJ4 female*)).mp.<br>***MENA***<br>exp Middle East/ or exp North Africa/ or exp Arab/ or exp Afghanistan/ or exp Djibouti/ or exp Pakistan/ or exp Somalia/ or exp Sudan/ or exp South Sudan/ or Middle East.mp. or North Africa.mp. or EMRO.mp. or Eastern Mediterranean.mp. or Arab.mp. or Arabs.mp. or Arab World.mp. or Islam.mp. or Afghanistan.mp. or Afghan*.mp. or Algeria*.mp. or Bahrain*.mp. or Djibouti.mp. or Egypt*.mp. or Jordan*.mp. or Kuwait*.mp. or Leban*.mp. or Libya*.mp. or Iran*.mp. or Iraq*.mp. or Morocc*.mp. or Oman*.mp. or Pakistan*.mp. or Qatar*.mp. or Saudi*.mp. or Somal*.mp. or Sudan*.mp. or Syria*.mp. or Tunisia*.mp. or United Arab Emirates.mp. or Emirat*.mp. or West Bank.mp. or Ghaza*.mp. or Gaza*.mp. or Palestin*.mp. or Yemen*.mp. or UAE.mp. or KSA.mp.<br>***Women***<br>exp female/ or (women or girl* or female*).mp.
**FINAL EMBASE SEARCH**<br>**“Sexually transmitted infections” AND “Sex work” AND “MENA” AND “Women”**
**Regional databases**
**Iran Scientific Information Database** (September 11, 2018**)**<br>Keyword search for: “chlamydia”, “gonorrhea”, “gonorrhaeae”, “gonococc”, “trichomonas”, “trichomoniasis”, “syphilis”, “treponema”, “HSV”, “herpes”, “venereal”, “sexually transmitted”, “pelvic inflammatory disease”, “reproductive tract infection”, “urinary tract infection”<br><br>**Iraq Academic Scientific Journals database** (September 10, 2018)<br>Keyword search for: “chlamydia”, “gonorrhea”, “gonorrhaeae”, “gonococc*”, “trichomonas”, “trichomoniasis”, “syphilis”, “treponema*”, “HSV”, “herpes”, “venereal”, “sexually transmitted”, “pelvic inflammatory disease”, “reproductive tract infection*”, “urinary tract infection*”<br><br>**MENA HIV/AIDS Epidemiology Synthesis Project database** (September 01, 2018)<br>Hand search of all documents in the database<br><br>**PakMediNet database** (September 12, 2018)<br>Keyword search for: “chlamydia”, “gonorrhea”, “gonorrhaeae”, “gonococcal”, “gonococcus”, “gonococci”, “trichomonas”, “trichomoniasis”, “syphilis”, “treponema”, “HSV”, “herpes”, “venereal”, “sexually transmitted”, “pelvic inflammatory disease”, “reproductive tract infection”, “urinary tract infection”<br><br>**World Health Organization Global Health Observatory data repository** (September 16, 2018)

Search by category: "sexually transmitted infections"<br><br>**World Health Organization African Index Medicus database** (September 08, 2018)<br>Keyword search for: "Algeria", "Algerie", "Djibouti", "Egypt", "Egypte", "Libya", "Libie", "Maroc", "Morocco", "Tunisia", "Tunisie", "Somalia", "Somalie", "Sudan", and "Soudan"<br><br>**World Health Organization Index Medicus for the Eastern Mediterranean Region database** (September 20, 2018)<br>Keyword search for: "chlamydia", "gonorrhea", "gonorrhaeae", "gonococcal", "gonococcus", "gonococci", "trichomonas", "trichomoniasis", "syphilis", "treponema", "HSV", "herpes", "venereal", "sexually transmitted", "pelvic inflammatory disease", "reproductive tract", "urinary tract"
**Abstract archives of the International AIDS Society conferences** (July 28, 2018)
Keyword search using each MENA country name

FSWs, female sex workers.

Box S2. List of extracted variables

Report characteristics
Author(s)
Year of publication
Full citation
Publication type
Data source

General study characteristics
Study population and its characteristics
Year(s) of data collection
Country of origin
Country of survey
City
Study site
Study design
Sampling methodology
Eligibility criteria
Participation rate

Sexually transmitted infection incidence
Number followed-up
Follow-up time
Seroconversion risk
Incidence rate
Specimen type (endocervical, urine, vaginal, serum)
Diagnostic method (polymerase chain reaction, culture, enzyme-linked immunoassay, rapid plasma reagin....)

Sexually transmitted infection prevalence
Number tested
Number positive
Specimen type (endocervical, urine, vaginal, serum)
Diagnostic method (polymerase chain reaction, culture, enzyme-linked immunoassay, rapid plasma reagin....)

Table S2. Definitions of types of infection and classification of results of diagnostic methods for *Treponema pallidum* (syphilis), *Chlamydia trachomatis*, *Neisseria gonorrhoeae*, *Trichomonas vaginalis*, and herpes simplex virus type 2 (HSV-2) in studies identified by the systematic review into current, recent, and ever infection.

**Infection**	**Current infection**	**Recent infection**	**Ever infection*** **(seropositivity using antibody testing)**
**Definition**	A state in which a person is currently a carrier of a pathogen responsible for causing the infection	A state in which a person has acquired the infection some time *recently*, but may or may not currently be a carrier of the pathogen	A state in which a person has acquired the infection some time in the past, but may or may not currently be a carrier of the pathogen
**STI**			
Treponema pallidum	Positive RPR test<br>Positive VDRL test<br>Positive RPR test or positive VRDL test with positive results confirmed through either positive FTA-ABS, or positive RDT, or positive TPHA tests	Not applicable	Positive FTA-ABS test<br>Positive RDT test<br>Positive TPHA test
Chlamydia trachomatis	Positive culture<br>Positive NAAT test<br>Positive immunofluorescence test on genital specimen (antigen detection)	Positive IgM serology<br>Positive IgA serology<br>Higher titers indicative of recent infection	Positive IgG serology
Neisseria gonorrhoeae	Positive culture<br>Positive NAAT test<br>Positive gram stain	Not applicable	Positive IgG serology
Trichomonas vaginalis	Positive culture<br>Positive wet mount<br>Positive NAAT test	Not applicable	Positive IgG serology
Herpes simplex virus type 2	Not applicable	Positive IgM serology<br>Positive IgA serology<br>Higher titers indicative of recent infection	Positive IgG serology

*Testing conducted for the total sample regardless of test results for current infection.
FTA-ABS, fluorescent treponemal antibody absorption test. IgG, immunoglobulin G. NAAT, nucleic acid amplification test. RDT, rapid diagnostic test. RPR, rapid plasma reagin. STI, sexually transmitted infection. TPHA, *Treponema pallidum* haemagglutination assay. VDRL, venereal disease research laboratory.

Table S3. Criteria for assessing the risk of bias (ROB) of *Treponema pallidum* (syphilis), *Chlamydia trachomatis*, *Neisseria gonorrhoeae*, *Trichomonas vaginalis*, and herpes simplex virus type 2 (HSV-2) prevalence studies among FSWs in the Middle East and North Africa (MENA), as identified by the systematic review

Quality domain	ROB assessment	Criteria
**1. Rigor of sampling methodology**	Low ROB	Studies using probability-based sampling
	High ROB	Studies using non-probability sampling
	Unclear	Information not reported
**2. Response rate**	Low ROB	≥60% or ≥60% of target sample size reached in studies using respondent-driven or time-location sampling
	High ROB	<60% or <60% of target sample size reached in studies using respondent-driven or time-location sampling
	Unclear	Information not reported
**3. Sexually transmitted infection ascertainment**	Low ROB	Biological assay for infection ascertainment explicitly indicated
	High ROB	--
	Unclear	Biological assay for infection ascertainment not explicitly indicated

FSWs, female sex workers.

Table S4. Details of independent variables included in the meta-regression analyses for syphilis prevalence

Variable	Sub-categories
**Country/subregion***	1. Eastern MENA: Afghanistan, Iran, and Pakistan<br>2. Egypt, Jordan, Yemen<br>3. North Africa: Algeria, Morocco, Sudan, and Tunisia<br>4. Horn of Africa: Djibouti, Somalia, and South Sudan
**Year of data collection**	Median
**Infection type**	1. Current<br>2. Ever (seropositivity using antibody testing)<br>3. Unclear
**Diagnostic method**	1. RPR/VDRL & TPHA/FTA-ABS/RDT<br>2. RPR/VDRL<br>3. TPHA<br>4. RDT<br>5. Not specified
**STI ascertainment**	1. Biological assay not explicitly reported<br>2. Biological assay explicitly indicated
**Sampling methodology**	1. Non-probability/unclear sampling<br>2. Probability-based sampling
**Sample size**	1. <100 participants<br>2. ≥100 participants
**Sampling methodology**	1. Non-probability/unclear sampling<br>2. Probability-based sampling such as respondent-driven sampling or systematic random sampling
**Response rate**	1. <60%/unclear<br>2. ≥60% or ≥60% of target sample size reached in studies using respondent-driven sampling or time-location sampling.

*Countries were grouped based on geography and similarity in prevalence levels.
FTA-ABS, fluorescent treponemal antibody absorption test. MENA, Middle East and North Africa. RDT, rapid diagnostic test. RPR, rapid plasma reagin. STI, sexually transmitted infection. TPHA, *Treponema pallidum* haemagglutination assay. VDRL, venereal disease research laboratory.

Table S5. Summary of the risk of bias (ROB) assessment for *Treponema pallidum* (syphilis), *Chlamydia trachomatis*, *Neisseria gonorrhea*, *Trichomonas vaginalis*, herpes simplex virus type 2 (HSV-2) prevalence studies among FSWs in the Middle East and North Africa (MENA)

ROB quality domains	Number of studies	%
**Rigor of sampling methodology**		
Low ROB	63	44.7
High ROB	65	46.1
Unclear	13	9.2
**Response rate**		
Low ROB	65	46.1
High ROB	3	2.1
Unclear	73	51.8
**Sexually transmitted infection ascertainment**		
Low ROB	111	78.7
High ROB	--	--
Unclear	30	21.3
**Total number of studies**	**141**[*]	**100.0**
**Summary**		
**Low ROB**		
At least 1 domain	120	85.1
At least 2 domains	80	56.7
All 3 domains	39	27.7
**High ROB**		
At least 1 domain	68	48.2
At least 2 domains	0	0
All 3 domains	0	0

[*]Three studies reported in the systematic review were excluded from further analyses, either because of the priority order followed for selecting studies applying the same assay to different biological specimens (2 studies), or because measures based on culture were superseded by measures based on polymerase chain reaction (1 study).
FSWs, female sex workers. NA, not applicable.

Table S6. Risk of bias (ROB) assessment for syphilis, *Chlamydia trachomatis*, *Neisseria gonorrhea*, *Trichomonas vaginalis*, herpes simplex virus type 2 (HSV-2) prevalence studies among FSWs in the Middle East and North Africa (MENA)

Country<br>Short citation*	Year(s) of data collection	Tested (n)	Prevalence	Sampling method	Response rate	STI ascertainment
**SYPHILIS CURRENT INFECTION**						
**Afghanistan**						
Todd, 2010 [2]	2006-08	520	0	High ROB	Unclear	Low ROB
**Egypt**						
MOH, 2000 [3]	1999-00	52	5.8	High ROB	Unclear	Low ROB
**Iran**						
Kassaian, 2012 [4]	2009-10	91	0	High ROB	Low ROB	Low ROB
Navadeh, 2012 [5]	2010	139	7.2	Low ROB	Low ROB	Low ROB
Kazerooni, 2014 [6]	2010-11	278	0	Low ROB	Low ROB	Low ROB
Jahanbakhsh, 2017 [7]	2012	14	0	High ROB	Unclear	Low ROB
**Morocco**						
MOH, 2008 [8]	2007	141	13.5	High ROB	Low ROB	Low ROB
MOH, 2012 [9]	2011-12	362	21.4	Low ROB	Low ROB	Low ROB
MOH, 2012 [9]	2011-12	359	18.8	Low ROB	Low ROB	Low ROB
MOH, 2012 [9]	2011-12	392	13.9	Low ROB	Low ROB	Low ROB
MOH, 2012 [9]	2011-12	318	13.3	Low ROB	Low ROB	Low ROB
**Pakistan**						
Baqi, 1998 [10]	1993-94	81*	5.0	High ROB	Low ROB	Low ROB
Rehan, 2009 [11] & NACP, 2005 [12]	2004	421	3.6	High ROB	Low ROB	Low ROB
Rehan, 2009 [11] & NACP, 2005 [12]	2004	387	16.0	Low ROB	Low ROB	Low ROB
Shah, 2004 [13]	2004	157	11.5	High ROB	Unclear	Low ROB
Hawkes, 2009 [14]	2007	107	2.8	Low ROB	Unclear	Low ROB
Hawkes, 2009 [14]	2007	426	1.2	Low ROB	Unclear	Low ROB
Khan, 2011 [15]	2007	730	4.5	Low ROB	Low ROB	Low ROB
**Somalia**						
Jama, 1987 [16]	1985-86	85	44.7	High ROB	Unclear	Low ROB
Jama Ahmed, 1991 [17]	1988-89	155	47.7	High ROB	Unclear	Low ROB
Scott, 1991 [18]	1989	57	50.8	High ROB	Unclear	Low ROB
Corwin, 1991 [19]	1990	302	35.4	High ROB	Unclear	Low ROB
Watts, 1994 [20]	1990	236	30.9	High ROB	Unclear	Low ROB
IOM, 2017 [21]	2014	96	2.4	Low ROB	High ROB	Low ROB
**Sudan**						
MOH, 2016 [22]	2015-16	832	7.3	Low ROB	Low ROB	Low ROB
**Tunisia**						
Bchir, 1988 [23]	1987	42	28.6	High ROB	Unclear	Low ROB
Ayachi, 1997 [24]	1992-94	79	24.1	High ROB	Unclear	Low ROB
**Yemen**						
Stulhofer, 2008 [25]	2008	244	4.9	Low ROB	Unclear	Low ROB
**SYPHILIS EVER INFECTION**†						
**Afghanistan**						
NACP, 2010 [26]	2009	368	5.4	Low ROB	Low ROB	Low ROB
NACP, 2012 [27]	2012	344	0.9	Low ROB	Low ROB	Low ROB
NACP, 2012 [27]	2012	333	0.0	Low ROB	Low ROB	Low ROB
NACP, 2012 [27]	2012	355	2.0	Low ROB	Low ROB	Low ROB
**Algeria**						
MOH, 2009 [28]	2004	185	11.9	High ROB	Unclear	Low ROB
MOH, 2009 [28]	2007	380	18.4	High ROB	Unclear	Low ROB
**Iran**						
Mirzazadeh, 2016 [29]	2015	1,337	0.4	High ROB	Unclear	Low ROB
**Pakistan**						
Hawkes, 2009 [14]	2007	107	2.8	Low ROB	Unclear	Low ROB
Hawkes, 2009 [14]	2007	426	1.6	Low ROB	Unclear	Low ROB
Bibi, 2010 [30]	2003	50	44.0	High ROB	Unclear	Low ROB
Raza, 2015 [31]	2014	NR	20.0	High ROB	Unclear	Low ROB
**Somalia**						

Jama, 1987 [16]	1985-86	85	57.6	High ROB	Unclear	Low ROB
Jama Ahmed, 1991 [17]	1988-89	155	69.0	High ROB	Unclear	Low ROB
Burans, 1990 [32]	NR	89	28.1	High ROB	Low ROB	Low ROB
IOM, 2017 [21]	2008	237	3.4	Low ROB	Low ROB	Low ROB
**Sudan**						
Sudan NACP, 2012 [33]	2011	305	1.5	Low ROB	Low ROB	Low ROB
Sudan NACP, 2012 [33]	2011	279	3.4	Low ROB	Low ROB	Low ROB
Sudan NACP, 2012 [33]	2011	282	3.4	Low ROB	Low ROB	Low ROB
Sudan NACP, 2012 [33]	2011	296	5.4	Low ROB	Low ROB	Low ROB
Sudan NACP, 2012 [33]	2011	288	4.3	Low ROB	Low ROB	Low ROB
Sudan NACP, 2012 [33]	2011	287	1.7	Low ROB	Low ROB	Low ROB
Sudan NACP, 2012 [33]	2011	303	5.2	Low ROB	Low ROB	Low ROB
Sudan NACP, 2012 [33]	2011	296	4.1	Low ROB	Low ROB	Low ROB
Sudan NACP, 2012 [33]	2011	293	8.9	Low ROB	Low ROB	Low ROB
Sudan NACP, 2012 [33]	2011	291	1.9	Low ROB	Low ROB	Low ROB
Sudan NACP, 2012 [33]	2011	303	5.3	Low ROB	Low ROB	Low ROB
Sudan NACP, 2012 [33]	2011	299	1.8	Low ROB	Low ROB	Low ROB
Sudan NACP, 2012 [33]	2011	284	1.8	Low ROB	Low ROB	Low ROB
Sudan NACP, 2012 [33]	2011	288	4.2	Low ROB	Low ROB	Low ROB
MOH, 2016 [22]	2015-16	832	12.0	Low ROB	Low ROB	Low ROB
**Tunisia**						
Bchir, 1988 [23]	1987	42	38.1	High ROB	Unclear	Low ROB
Ayachi, 1997 [24]	1992-94	79	36.7	High ROB	Unclear	Low ROB
Znazen, 2010 [34]	2007	183	2.7	High ROB	Low ROB	Low ROB
**SYPHILIS UNCLEAR**						
**Afghanistan**						
WHO, 2018 [35]	2010	NR	8.7	Unclear	Unclear	Unclear
MENA HIV ESP, 2013 [36]	2012	440	5.7	Unclear	Unclear	Unclear
WHO, 2018 [35]	2017	2,457	1.3	Unclear	Unclear	Unclear
**Algeria**						
WHO, 2018 [35]	2013	27	7.4	Unclear	Unclear	Unclear
WHO, 2018 [35]	2014	24	29.2	Unclear	Unclear	Unclear
WHO, 2018 [35]	2016	183	14.2	High ROB	Unclear	Unclear
WHO, 2018 [35]	2017	81	16.0	High ROB	Unclear	Unclear
**Djibouti**						
WHO, 2015 [37]	2014	361	5.0	Unclear	Unclear	Unclear
**Iran**						
WHO, 2018 [35]	2008	NR	1.6	Unclear	Unclear	Unclear
Moayedi-Nia, 2016 [38]	2012-13	161	0	Low ROB	Unclear	Unclear
**Jordan**						
WHO, 2015 [37]	2008	NR	6.7	Unclear	Unclear	Unclear
**Morocco**						
Khattabi, 2005 [39]	2004	332	9.6	High ROB	Unclear	Unclear
Khattabi, 2005 [39]	2004	272	12.1	High ROB	Unclear	Unclear
Khattabi, 2005 [39]	2004	143	9.0	High ROB	Unclear	Unclear
Bennani, 2006 [40]	2005	102	11.8	High ROB	Unclear	Unclear
Bennani, 2006 [40]	2005	143	13.3	High ROB	Unclear	Unclear
WHO, 2018 [35]	2008	NR	16.9	Unclear	Unclear	Unclear
**Pakistan**						
MENA HIV ESP, 2010 [41]	2007	NR	23.5	Unclear	Unclear	Unclear
**Somalia**						
WHO, 2018 [35]	2017	860	2.7	Low ROB	Unclear	Unclear
**Sudan**						
WHO, 2018 [35]	2016	4,123	4.1	Low ROB	Unclear	Unclear
WHO, 2018 [35]	2017	1,244	14.4	Unclear	Unclear	Unclear
**Yemen**						
WHO, 2018 [35]	2010	301	0	Low ROB	Unclear	Unclear
**CHLAMYDIA TRACHOMATIS CURRENT INFECTION**						
**Algeria**						
Kadi, 1989 [42]	NR	44	45.5	High ROB	Unclear	Low ROB
**Egypt**						
MOH, 2000 [3]	1999-00	52	7.7	High ROB	Unclear	Low ROB

**Iran**						
Darougar, 1983 [43]	NR	116	6.9	High ROB	Unclear	Low ROB
Kazerooni, 2014 [6]	2010-11	278	9.0	Low ROB	Low ROB	Low ROB
Mirzazadeh, 2016 [29]	2015	1,337	6.0	High ROB	Unclear	Low ROB
**Morocco**						
MOH, 2008 [8]	2007	141	22.7	High ROB	Low ROB	Low ROB
MOH, 2012 [9]	2011-12	368	22.4	Low ROB	Low ROB	Low ROB
**Pakistan**						
Rehan, 2009 [11]	2004	348	5.2	High ROB	Low ROB	Low ROB
Rehan, 2009 [11]	2004	383	11.0	Low ROB	Low ROB	Low ROB
Hawkes, 2009 [14]	2007	107	0.9	Low ROB	Unclear	Low ROB
Hawkes, 2009 [14]	2007	426	1.7	Low ROB	Unclear	Low ROB
Khan, 2011 [15]	2007	730	7.7	Low ROB	Low ROB	Low ROB
**Somalia**						
IOM, 2017 [21]	2014	90	0.7	Low ROB	High ROB	Low ROB
**Tunisia**						
Znazen, 2010 [34]	2007	188	72.9	Low ROB	High ROB	Low ROB
**CHLAMYDIA TRACHOMATIS RECENT INFECTION**						
**Algeria**						
Kadi, 1989 [42]	NR	44	95.0	High ROB	Unclear	Low ROB
**Iran**						
Darougar, 1983 [43]	NR	154	29.2	High ROB	Unclear	Low ROB
**CHLAMYDIA TRACHOMATIS EVER INFECTION**[†]						
**Algeria**						
Kadi, 1989 [42]	NR	44	100	High ROB	Unclear	Low ROB
**Iran**						
Darougar, 1983 [43]	NR	154	94.2	High ROB	Unclear	Low ROB
Kassaian, 2012 [4]	2009-10	91	19.8	High ROB	Low ROB	Low ROB
**Tunisia**						
Bchir, 1988 [23]	1987	42	73.8	High ROB	Unclear	Low ROB
Znazen, 2010 [34]	2007	183	85.8	High ROB	Low ROB	Low ROB
**CHLAMYDIA TRACHOMATIS UNCLEAR**						
**Iran**						
Navadeh, 2012 [5] & WHO, 2011 [44]	2010	144	2.9	Low ROB	Low ROB	Unclear
**Morocco**						
MENA HIV ESP, 2010 [41]	NR	NR	19.1	Unclear	Unclear	Unclear
**NEISSERIA GONORRHOEAE CURRENT INFECTION**						
**Egypt**						
MOH, 2000 [3]	1999-00	52	7.7	High ROB	Unclear	Low ROB
**Iran**						
Kazerooni, 2014 [6]	2010-11	278	1.4	Low ROB	Low ROB	Low ROB
Navadeh, 2012 [5] & WHO, 2011 [44]	2010	144	0	Low ROB	Low ROB	Unclear
Nasirian, 2017 [45]	2013-14	99	9.1	High ROB	Low ROB	Low ROB
Taghizadeh, 2015 [46]	2014	117	1.0	High ROB	Low ROB	Unclear
Mirzazadeh, 2016 [29]	2015	1,337	1.3	High ROB	Unclear	Low ROB
**Morocco**						
MOH, 2008 [8]	2007	141	10.6	High ROB	Low ROB	Low ROB
MENA HIV ESP, 2010 [41]	NR	NR	3.5	Unclear	Unclear	Unclear
MOH, 2012 [9]	2011-12	368	11.7	Low ROB	Low ROB	Low ROB
**Pakistan**						
Rehan, 2009 [11]	2004	348	9.8	High ROB	Low ROB	Low ROB
Rehan, 2009 [11]	2004	383	12.3	Low ROB	Low ROB	Low ROB
Hawkes, 2009 [14]	2007	107	1.9	Low ROB	Unclear	Low ROB
Hawkes, 2009 [14]	2007	426	2.0	Low ROB	Unclear	Low ROB
Khan, 2011 [15]	2007	730	7.5	Low ROB	Low ROB	Low ROB
**Somalia**						
Burans, 1990 [32]	NR	89	11.2	High ROB	Low ROB	Low ROB
IOM, 2017 [21]	2014	91	0.4	Low ROB	High ROB	Low ROB
**Tunisia**						
NACP, 2005 [47]	2005	NR	12.0-17.0[‡]	High ROB	Unclear	Unclear
Znazen, 2010 [34]	2007	188	11.2	High ROB	Low ROB	Low ROB

**TRICHOMONAS VAGINALIS CURRENT INFECTION**						
**Egypt**						
MOH, 2000 [3]	1999-00	52	19.2	High ROB	Unclear	Low ROB
**Iran**						
Vafaei, 2015 [48]	2009-11	85	8.2	High ROB	Low ROB	Low ROB
Navadeh, 2012 [5] & WHO, 2011 [44]	2010	144	1.4	Low ROB	Low ROB	Unclear
Nasirian, 2017 [45]	2013-14	99	0.0	High ROB	Low ROB	Low ROB
Mirzazadeh, 2016 [29]	2015	1,337	11.9	High ROB	Unclear	Low ROB
**Morocco**						
MOH, 2008 [8]	2007	141	14.9	High ROB	Low ROB	Low ROB
MOH, 2012 [9]	2011-12	367	11.8	Low ROB	Low ROB	Low ROB
**Pakistan**						
Rehan, 2009 [11]	2004	386	5.2	High ROB	Low ROB	Low ROB
Rehan, 2009 [11]	2004	384	19.3	Low ROB	Low ROB	Low ROB
Hawkes, 2009 [14]	2007	107	5.7	Low ROB	Unclear	Low ROB
Hawkes, 2009 [14]	2007	426	4.3	Low ROB	Unclear	Low ROB
Khan, 2011 [15]	2007	730	5.1	Low ROB	Low ROB	Low ROB
**HSV-2 EVER INFECTION†**						
**Pakistan**						
Hawkes, 2009 [14]	2007	107	4.7	Low ROB	Unclear	Low ROB
Hawkes, 2009 [14]	2007	426	8.0	Low ROB	Unclear	Low ROB
**Syria**						
Ibrahim, 2000 [49]	1995-98	101	22.8	High ROB	Unclear	Low ROB
Ibrahim, 2000 [49]	1995-98	125	20.0	High ROB	Unclear	Low ROB
**Tunisia**						
Znazen, 2010 [34]	2007	183	55.5	High ROB	Low ROB	Low ROB

The table is sorted, for each country, by data collection year(s) then city/province.

*Three studies reported in the systematic review were excluded from further analyses, either because of the priority order followed for selecting studies applying the same assay to different biological specimens (2 studies), or because measures based on culture were superseded by measures based on polymerase chain reaction (1 study).

†Ever infection indicates seropositivity using antibody testing.

‡Range reported based on several studies whose abstracts or full-texts could not be retrieved (mid-point: 14.5%).

IOM, International Organization for Migration. MENA HIV ESP, MENA HIV/AIDS Epidemiology Synthesis Project database. MOH, Ministry of Health. NACP, National AIDS Control Program. NR, not reported. STI, sexually transmitted infection. WHO, World Health Organization.

Table S7. Results of meta-analyses stratified by subregion on prevalence studies for current and ever infection with *Treponema pallidum* (syphilis) among FSWs in the Middle East and North Africa

Sexually transmitted infection	Studies	Samples		Reported prevalence		Pooled mean prevalence		Heterogeneity measures		
	N	Tested	Positive	Median* (%)	Range* (%)	Estimate (%)	95% CI	Q† (p-value)	I^{2}‡ (%; 95% CI)	Prediction interval§ (95%)
**Subregion**										
**Current infection**										
Eastern MENA	13	3,351	150	3.6	0-16.0	3.0	0.9-9.2	203.7 (p<0.0001)	94.1 (91.5-95.9)	0.0-20.9
Egypt, Jordan, and Yemen	2[ǁ]	296	15	5.4	4.9-5.8	--	--	--	--	--
North Africa	7	1,693	293	18.8	13.3-28.6	17.6	14.2-21.3	19.1 (p=0.004)	68.6 (30.7-85.8)	8.3-29.5
Horn of Africa	12	1,763	384	32.9	2.4-62.0	27.8	15.2-42.4	350.1 (p<0.0001)	96.9 (95.7-97.7)	0.0-84.4
**Ever infection**[¶]										
Eastern MENA	9	3,604	125	2.0	0-44.0	4.6	1.3-9.7	250.5 (p<0.0001)	96.8 (95.4-97.8)	0.0-30.3
Egypt, Jordan, and Yemen	0	--	--	--	--	--	--	--	--	--
North Africa	30	4,963	297	5.3	0-38.1	7.7	5.4-10.4	267.2 (p<0.0001)	89.1 (85.6-91.8)	0.0-25.0
Horn of Africa	11	1,401	288	52.5	3.1-92.3	46.8	26.6-67.4	388.5 (p<0.0001)	97.4 (96.5-98.1)	0.0-67.4

The same population may have contributed different measures for both current infection and ever (seropositivity using antibody testing) infection.

*Medians and ranges were calculated based on the stratified prevalence measures.

†Q: the Cochran's Q statistic is a measure assessing the existence of heterogeneity in effect size (here, prevalence) across studies.

‡I^2: a measure assessing the magnitude of between-study variation that is due to differences in effect size (here, prevalence) across studies rather than chance.

§Prediction interval: a measure estimating the 95% interval of the distribution of true effect sizes (here, prevalence measures).

ǁMeta-analyses were performed if at least three studies were available.

¶Ever infection indicates seropositivity using antibody testing.

CI, confidence interval. FSWs, female sex workers.

Table S8. Results of stratified meta-analyses by year of data collection on prevalence studies for current and ever infection with *Treponema pallidum* (syphilis) and current infection with *Chlamydia trachomatis*, *Neisseria gonorrhoeae*, and *Trichomonas vaginalis* among FSWs in the Middle East and North Africa

**Sexually transmitted infection**	**Studies**	**Samples**		**Reported prevalence**		**Pooled mean prevalence**		**Heterogeneity measures**		
	N	Tested	Positive	Median[*] (%)	Range[*] (%)	Estimate (%)	95% CI	Q[†] (p-value)	I^2[‡] (%; 95% CI)	Prediction interval[§] (95%)
***Treponema pallidum* (syphilis)**										
**Current infection**										
<2010	25	4,313	526	11.5	0-62.0	15.0	8.8-22.3	855.1 (p<0.0001)	97.2 (96.6-97.7)	0.0-60.0
≥2010	9	2,790	316	7.3	0-21.4	8.0	3.4-14.1	187.3 (p<0.0001)	95.7 (93.6-97.1)	0.0-35.8
**Ever infection**[ǀ]										
<2010	30	2,386	382	29.2	0-92.3	24.6	16.1-34.2	693.4 (p<0.0001)	95.8 (94.8-96.6)	0.0-80.2
≥2010	20	7,582	328	3.4	0-20.0	3.6	2.0-5.6	342.2 (p<0.0001)	94.4 (92.6-95.8)	0.0-16.9
Chlamydia trachomatis										
**Current infection**										
<2010	12	2,535	325	8.8	0.9-76.2	17.1	7.9-28.8	526.1 (p<0.0001)	97.9 (97.3-98.4)	0.0-69.9
≥2010	4	2,073	187	7.5	0.7-22.4	8.4	2.4-17.3	80.6 (p<0.0001)	96.3 (93.1-98.0)	0.0-65.1
Neisseria gonorrhoeae										
**Current infection**										
<2010	13	2,796	227	9.7	1.9-17.5	8.1	5.6-10.9	73.2 (p<0.0001)	83.6 (73.4-89.9)	0.9-20.6
≥2010	7	2,434	74	1.3	0-11.7	2.2	0.2-5.8	88.0 (p<0.0001)	93.2 (88.4-96.0)	0.0-20.6
Trichomonas vaginalis										
**Current infection**										
<2010	8	2,226	186	6.4	1.2-19.3	8.2	4.2-13.3	100.9 (p<0.0001)	93.1 (88.6-95.8)	0.0-30.6
≥2010	5	2,032	211	8.2	0-11.9	5.5	1.6-11.2	56.8 (p<0.0001)	93.0 (86.5-96.3)	0.0-35.0

The same population may have contributed different measures for both current infection and ever (seropositivity using antibody testing) infection.
[*]Medians and ranges were calculated based on the stratified prevalence measures.
[†]Q: the Cochran's Q statistic is a measure assessing the existence of heterogeneity in effect size (here, prevalence) across studies.
[‡]I^2: a measure assessing the magnitude of between-study variation that is due to differences in effect size (here, prevalence) across studies rather than chance.
[§]Prediction interval: a measure estimating the 95% interval of the distribution of true effect sizes (here, prevalence measures).
[ǀ]Ever infection indicates seropositivity using antibody testing.
CI, confidence interval. FSWs, female sex workers.

Figure S1. Scatter plots showing the time trend for the prevalence of A) current and B) ever infection with *Treponema pallidum* (syphilis) among FSWs in the Middle East and North Africa

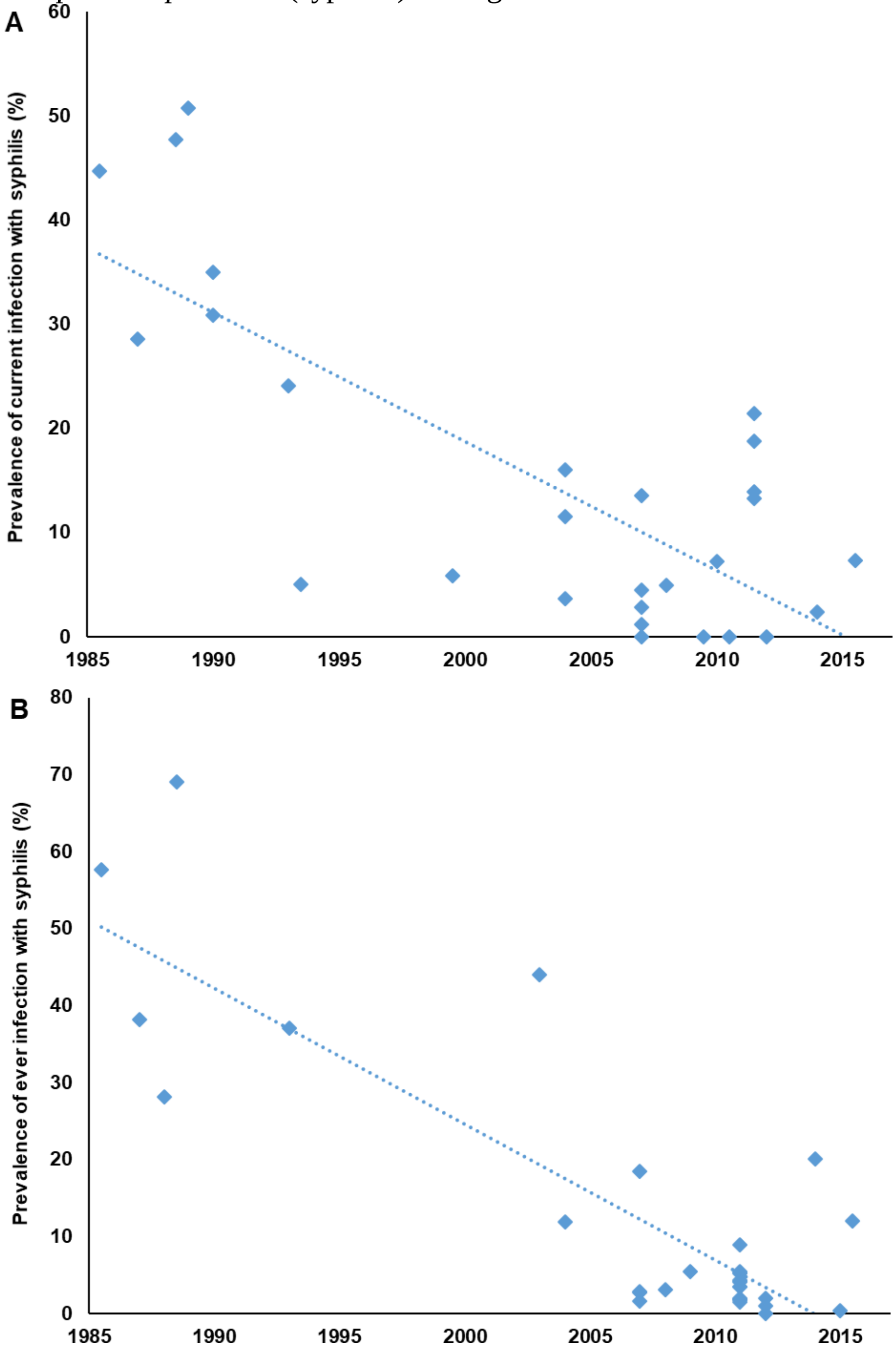

References

1 Moher D, Liberati A, Tetzlaff J, Altman DG, Group P. Preferred reporting items for systematic reviews and meta-analyses: The PRISMA statement. PLoS medicine. 2009;6:e1000097.

2 Todd CS, Nasir A, Stanekzai MR, Bautista CT, Botros BA, Scott PT, et al. HIV, hepatitis B, and hepatitis C prevalence and associated risk behaviors among female sex workers in three Afghan cities. Aids. 2010;24 Suppl 2:S69-75.

3 Ministry of Health and Population, National AIDS Program. Evaluation of selected reproductive health infections in various Egyptian population groups in Greater Cairo. Cairo, Egypt: 2000.

4 Kassaian N, Ataei B, Yaran M, Babak A, Shoaei P, Ataie M. HIV and other sexually transmitted infections in women with illegal social behavior in Isfahan, Iran. Adv Biomed Res. 2012;1:5.

5 Navadeh S, Mirzazadeh A, Mousavi L, Haghdoost A, Fahimfar N, Sedaghat A. HIV, HSV2 and syphilis prevalence in female sex workers in Kerman, South-East Iran; using respondent-driven sampling. Iran J Public Health. 2012;41:60-5.

6 Kazerooni PA, Motazedian N, Motamedifar M, Sayadi M, Sabet M, Lari MA, et al. The prevalence of human immunodeficiency virus and sexually transmitted infections among female sex workers in Shiraz, south of Iran: By respondent-driven sampling. International Journal of STD and AIDS. 2014;25:155-61.

7 Jahanbakhsh F, Bagheri Amiri F, Sedaghat A, Fahimfar N, Mostafavi E. Prevalence of HAV Ab, HEV (IgG), HSV2 IgG, and syphilis among sheltered homeless adults in Tehran, 2012. Int J Health Policy Manag. 2017;7:225-30.

8 Royaume du Maroc-Ministere de la Sante. Etude de prevalence des IST chez les femmes qui consultent pour pertes vaginales et/ou douleurs du bas ventre. Rabat, Maroc: Programme National de lutte contre les IST/SIDA, 2008.

9 Ministry of Health-Morocco, The Joint United Nations Programme on HIV/AIDS (UNAIDS), The Global Fund. HIV integrated behavioral and biological surveillance surveys-Morocco 2011: Female sex workers in Agadir, Fes, Rabat and Tanger. Morocco: 2012.

10 Baqi S, Nabi N, Hasan SN, Khan AJ, Pasha O, Kayani N, et al. HIV antibody seroprevalence and associated risk factors in sex workers, drug users, and prisoners in Sindh, Pakistan. Journal of acquired immune deficiency syndromes and human retrovirology : official publication of the International Retrovirology Association. 1998;18:73-9.

11 Rehan N, Bokhari A, Nizamani NM, Jackson D, Naqvi HR, Qayyum K, et al. National study of reproductive tract infections among high risk groups of Lahore and Karachi. Journal of the College of Physicians and Surgeons Pakistan. 2009;19:228-31.

12 Ministry of Health-Pakistan National AIDS Control Program. National study of reproductive tract and sexually transmitted infections: A survey of high risk groups in Lahore and Karachi, Pakistan. 2005.

13 Shah A.S., Memon M.A., Soomro S., Kazi N., Kristensen S., editors. Seroprevelance of HIV, syphilis, hepatitis B and hepatitis C among female commercial sex workers in Hyderabad, Pakistan. International AIDS Conference, C12368; 2004.

14 Hawkes S, Collumbien M, Platt L, Lalji N, Rizvi N, Andreasen A, et al. HIV and other sexually transmitted infections among men, transgenders and women selling sex in two cities in Pakistan: A cross-sectional prevalence survey. Sexually Transmitted Infections. 2009;85:ii8-ii16.

15 Khan MS, Unemo M, Zaman S, Lundborg CS. HIV, STI prevalence and risk behaviours among women selling sex in Lahore, Pakistan. BMC Infectious Diseases. 2011;11 (no pagination).

16 Jama H, Hederstedt B, Osman S, Omar K, Isse A, Bygdeman S. Syphilis in women of reproductive age in Mogadishu, Somalia: Serological survey. Genitourinary Medicine. 1987;63:326-8.

17 Jama Ahmed H, Omar K, Adan SY, Guled AM, Grillner L, Bygdeman S. Syphilis and human immunodeficiency virus seroconversion during a 6-month follow-up of female prostitutes in Mogadishu, Somalia. International Journal of STD and AIDS. 1991;2:119-23.

18 Scott DA, Corwin AL, Constantine NT, Omar MA, Guled A, Yusef M, et al. Low prevalence of human immunodeficiency virus-1 (HIV-1), HIV-2, and human T cell lymphotropic virus-1 infection in Somalia. American Journal of Tropical Medicine and Hygiene. 1991;45:653-9.

19 Corwin AL, Olson JG, Omar MA, Razaki A, Watts DM. HIV-1 in Somalia: Prevalence and knowledge among prostitutes. Aids. 1991;5:902-4.

20 Watts DM, Corwin AL, Omar MA, Hyams KC. Low risk of sexual transmission of hepatitis C virus in Somalia. Transactions of the Royal Society of Tropical Medicine and Hygiene. 1994;88:55-6.

21 International Organization for Migration (IOM). Integrated biological and behavioural surveillance survey among vulnerable women in Hargeisa, Somaliland. Geneva, Switzerland: 2017.

22 Government of the Republic of South Sudan-Ministry of Health. A bio-behavioral HIV survey of female sex workers in South Sudan. South Sudan: 2016.

23 Bchir A, Jemni L, Saadi M, Milovanovic A, Brahim H, Catalan F. Markers of sexually transmitted diseases in prostitutes in central Tunisia. Genitourinary medicine. 1988;64:396-7.

24 Ayachi F, Kechrid A, Lagha N, Ben Hamida A, Amamou H, Ben Mahmoud R. Seroprevalence rate of syphilis in 3 groups of sexually active tunisian women. [French]. Medecine et Maladies Infectieuses. 1997;27:913-4.

25 Stulhofer A, Bozicevic I. HIV bio-behavioural survey among female sex workers in Aden, Yemen. 2008.

26 National AIDS Control Program, Johns Hopkins University Bloomberg School of Public Health HIV Surveillance Project. Integrated behavioral & biological surveillance (IBBS) in Afghanistan: Year 1 report. Kabul, Afghanistan: 2010.

27 National AIDS Control Program, Johns Hopkins University Bloomberg School of Public Health HIV Surveillance Project. Integrated biological & behavioral surveillance (IBBS) in selected cities of Afghanistan: Findings of 2012 IBBS survey and comparison to 2009 IBBS survey. Kabul, Afghanistan: 2012.

28 Ministere de la Sante et de la Population et de la Reforme Hospitaliere, Direction de la Prevention Comite National de Lutte contre les IST/VIH/SIDA. Plan national strategique de lutte contre les IST/VIH/Sida 2008-2012. Geneva, Switzerland: 2009.

29 Mirzazadeh A, M. Shokoohi, R. Khajehkazemi, et al., editor HIV and sexually transmitted infections among female sex workers in Iran: Findings from the 2010 and 2015 national surveillance surveys. 21st International AIDS Conference, Durban, South Africa, 7/18-22, ePoster, Abstract TUPEC175; 2016.

30 Bibi I, Devrajani BR, Shah SZA, Soomro MH, Jatoi MA. Frequency of syphilis in female sex workers at red light area of Hyderabad, Pakistan. Journal of the Pakistan Medical Association. 2010;60:353-6.

31 Raza M, Ikram N, Saeed N, Waheed U, Kamran M, Iqbal R, et al. HIV/AIDS and syphilis screening among high risk groups. J Rawal Med Coll. 2015;19:11-4.

32 Burans JP, Fox E, Omar MA, Farah AH, Abbass S, Yusef S, et al. HIV infection surveillance in Mogadishu, Somalia. East African medical journal. 1990;67:466-72.

33 Sudan National AIDS Control Program. Integrated bio-behavioral HIV surveillance (IBBS) among female sex workers and men who have sex with men in 15 states of Sudan, 2011-2012. 2012.

34 Znazen A, Frikha-Gargouri O, Berrajah L, Bellalouna S, Hakim H, Gueddana N, et al. Sexually transmitted infections among female sex workers in Tunisia: High prevalence of *Chlamydia trachomatis*. Sexually Transmitted Infections. 2010;86:500-5.

35 World Health Organization. Global health observatory data repository. 2018. Available: http://apps.who.int/gho/data/node.main.A1360STI?lang=en. Accessed.
36 Additional country-level data provided through the MENA HIV/AIDS Epidemiology Synthesis Project database by the World Health Organization Regional Office for the Eastern Mediterranean. 2013.
37 World Health Organization. Report on globally sexually transmitted infection surveillance 2015. Geneva, Switzerland: World Health Organization, 2016 Contract No.: ISBN 978 92 4 156530 1
38 Moayedi-Nia S, Bayat Jozani Z, Esmaeeli Djavid G, Entekhabi F, Bayanolhagh S, Saatian M, et al. HIV, HCV, HBV, HSV, and syphilis prevalence among female sex workers in Tehran, Iran, by using respondent-driven sampling. AIDS Care. 2016;28:487-90.
39 Khattabi H., Alami K. Surveillance sentinelle du VIH: Resultats 2004 et tendances de la seroprevalence du VIH. Morocco: 2005.
40 Bennani A., Alami K. Surveillance sentinelle du VIH: Resultats 2005 et tendances de la seroprevalence du VIH. 2006.
41 Abu-Raddad L, Akala FA, Semini I, Riedner G, Wilson D, Tawil O. Characterizing the HIV/AIDS epidemic in the Middle East and North Africa: Time for strategic action. World Bank/UNAIDS/WHO Publication, editor. Washington DC: The World Bank Press; 2010.
42 Kadi Z, Bouguermouh A, Ait-Mokhtar N, Allouache A, Ziat A, Orfilla J. Genital chlamydia infections. A seroepidemiologic study in Algiers. [French]. Archives de l'Institut Pasteur d'Algerie Institut Pasteur d'Algerie. 1989;57:73-82.
43 Darougar S, Aramesh B, Gibson JA, Treharne JD, Jones BR. Chlamydial genital infection in prostitutes in Iran. British Journal of Venereal Diseases. 1983;59:53-5.
44 World Health Organization. HIV surveillance systems: Regional update 2011. 2011.
45 Nasirian M, Kianersi S, Hoseini SG, Kassaian N, Yaran M, Shoaei P, et al. Prevalence of sexually transmitted infections and their risk factors among female sex workers in Isfahan, Iran: A cross-sectional study. Journal of the International Association of Providers of AIDS Care. 2017;16:608-14.
46 Taghizadeh H, Taghizadeh F, Fathi M, Reihani P, Shirdel N, Rezaee SM. Drug use and high-risk sexual behaviors of women at a drop-in center in mazandaran province, Iran, 2014. Iranian Journal of Psychiatry and Behavioral Sciences. 2015;9:49-55.
47 Programme de Lutte contre les IST/SIDA. Analyse de la situation et de la reponse au VIH/SIDA en Tunisie. Tunisia: 2005.
48 Vafaei H., Asadi N., Foroughinia L., Salehi A., Kuhnavard S., Akbarzadeh M., et al. Comparison of abnormal cervical cytology from HIV positive women, female sex workers, and general population. IJCBNM. 2015;3:76-83.
49 Ibrahim AI, Kouwatli KM, Obeid MT. Frequency of herpes simplex virus in Syria based on type-specific serological assay. Saudi medical journal. 2000;21:355-60.
